# Aerobiology in Africa: a continent-wide review of pollen, fungal spores, and allergy risk

**DOI:** 10.1007/s10453-026-09933-w

**Published:** 2026-07-17

**Authors:** Dorra Gharbi, Linus Ajikah, Keneilwe Podile, Niké Susan Wesch, Abdelkader Amroune, Ijlal Raissouni, Asmaa Boullayali, Hery Lisy Tiana Ranarijoana, Estelle Razanatsoa, Fanirisoa Razafiarison, Monique Tossou, Karabo Makgaretse, Frank Harald Neumann

**Affiliations:** 1 Unit for Environmental Sciences and Management, Faculty of Natural and Agricultural Science, North-West University, Potchefstroom, South Africa; 2 Analysis and Experimentation On Ecosystem, CNRS Campus, Gif-Sur-Yvette, Paris, France; 3 Department of Plant and Ecological Studies, (PES) Faculty of Biological Science, University of Calabar, Calabar, Nigeria; 4 Evolutionary Studies Institute, (ESI) University of the Witwatersrand Johannesburg South Africa, Johannesburg, South Africa; 5 Medical Botany Laboratory, Department of Pharmacy, Faculty of Medicine, University of Batna 2, Batna, Algeria; 6 Laboratory of Biology, Ecology and Health, Applied Botany Team, Department of Biology, Faculty of Sciences, University Abdelmalek Essaâdi Mhannech II, Tétouan, Morocco; 7 Ecole Doctorale Ecosystèmes Naturels (EDEN), University of Mahajanga, Mahajanga, Madagascar; 8 Department of Biological Sciences, University of Cape Town, Cape Town, South Africa; 9 Laboratory of Vegetal Sciences and Pharmacopoeia (LaSVEP), University of Abomey-Calavi, Abomey-Calavi, Benin; 10 Centre for Global Change, North-West University, Potchefstroom, South Africa

**Keywords:** Africa, Aerobiology, Aerospora, Environmental studies, Public health

## Abstract

Aerobiology is pivotal for understanding human health risks, ecosystem dynamics, as well as plant reproduction, including phylogeny. Africa’s vast climatic and ecological diversity—from deserts to tropical rainforests—produces complex, region-specific aerobiological patterns that are often underinvestigated. Seasonal and meteorological factors, including rainfall, temperature, humidity, wind, and phenomena such as the Harmattan, significantly influence the distribution of airborne pollen and fungal spores, with direct implications for allergen exposure and the development of respiratory diseases. These complex systems are altered by anthropogenic climate change and disturbances. Despite these impacts, continent-wide aerobiological research remains limited, with most studies concentrated in research-strong nations such as South Africa and Nigeria. This review synthesizes current knowledge on African aerospora, highlighting spatial and temporal trends, dominant taxa, environmental drivers, sampling methodologies, and links to allergic disease, including projections under climate change. Data were collated from PubMed, Web of Science, Scopus, African Journals Online, and long-term monitoring initiatives. Key research gaps include sparse long-term monitoring data, inconsistent methodologies, and limited integration with public health outcomes and modelling approaches. Expanding monitoring networks and fostering interdisciplinary approaches are essential for improving our understanding of African aeroallergens and informing strategies to mitigate allergy and respiratory health risks, such as inhibiting the spread of invader plants such as *Ambrosia* and planting of exotic trees that produce an abundance of highly allergenic pollen, such as *Morus* and *Platanus*.

## Introduction

Aerobiology is a multidisciplinary field that bridges ecology, meteorology, and public health. Aerospora transport pathways and dispersal influence not only ecosystem processes, such as phylogeny, seed set, and gene flow, but also human health, contributing substantially to seasonal allergic diseases, including allergic rhinitis and asthma, as well as associated economic impacts (Moitra & Kavitha, 2024). Their concentrations and composition are highly influenced by vegetation cover, climate variability, anthropogenic impacts, and seasonal phenology, making aerobiological studies essential for understanding ecological processes also in agriculture, predicting allergenic risk, and informing public health strategies. In this context, Van Leuken et al. (2016) aimed to assess how modelled climate change may alter aerospora levels, dispersal, and phenology by influencing meteorological factors such as temperature, rainfall, and wind speed.

Aerobiological research is highly advanced in Europe, North America, and Eastern Asia compared to other parts of the world, with several long-standing national and continental monitoring networks that systematically detect airborne pollen and fungal spores (Dwarakanah et al., 2024; Lucas & Bunderson, 2024). National and regional aerobiological monitoring networks, e.g. Germany’s PID, France’s RNSA, and Japan’s automated cedar pollen monitoring systems, have utilized Hirst-type volumetric traps since the 1970s–1990s to collect continuous, standardized data (Buters et al., 2018) and increasingly rely on automated spore traps (Oteros et al., 2019; Clot et al., 2024; Tummon et al., 2024; Sofiev et al., 2023; Farooq et al., 2025). In contrast, North American programmes, including the National Allergy Bureau, historically used Durham gravimetric samplers extensively before progressively incorporating volumetric methods (Durham, 1946). Australia’s AusPollen Partnership, officially established in 2016, has further expanded standardized airborne pollen surveillance worldwide (Emmerson et al., 2022). These networks provide detailed pollen calendars, track seasonal and interannual variations, and integrate meteorological data to understand dispersal patterns.

In comparison, aerobiological research in Africa has few long-term monitoring networks and limited standardized data, meaning that comprehensive pollen calendars, continuous Hirst-type sampling, and continent-wide allergy forecasting are less developed (Ajikah et al., 2020, 2021). The strong bias towards the Global North, but also recent progress in the Global South, is best illustrated when visiting the global map of pollen monitoring sites, Worldwide Pollen Map—EAACI (Buters et al., 2018).

Systematic and continent-wide aerobiological studies remain limited, while increasingly well-documented public health impacts of aeroallergens are being recorded. The majority of aerobiological research in Africa has been conducted in South Africa and Nigeria—countries with strong research networks, leaving vast regions of the continent underrepresented in the literature (Ajikah et al., 2020, 2021). Studies indicate that fungal spores such as *Alternaria*, *Cladosporium*, and *Curvularia*, alongside pollen from Poaceae (grasses) and various tree taxa, including many ornamental and plantation trees introduced from Europe, North America, Asia, and Australia, are prevalent in urban and rural environments (Ajikah et al., 2020; Esterhuizen et al., 2023; Gharbi et al., 2023a, 2023b). A study in Potchefstroom, northwestern South Africa, linked airborne aeroallergen profiles with sensitization patterns among adults as a result of pollen allergy skin prick tests, reporting high rates of sensitization to grass pollen (e.g. *Cynodon dactylon*) and fungal spores (particularly *Alternaria*). Statistically significant correlations exist between aeroallergen concentrations and allergic rhinitis symptoms (Gharbi et al., 2025b). These data underscore the public health relevance of aerobiological surveillance and highlight the need for comprehensive allergy testing integrated with aerobiological monitoring in understudied and under-resourced regions where public health infrastructure is lacking (Mbugi & Chilongola, 2010). In South Africa, local rainfall, temperature, relative humidity, and wind dynamics have been shown to explain aerospora variability, clear seasonal patterns, and implications for increased allergen exposure during specific high-risk periods (Ajikah et al., 2023; Roffe et al., 2025).

Critical research gaps remain. Long-term monitoring networks are scarce, especially in West, Central, East, and North Africa, and aerobiological data are poorly integrated with public health outcomes, skin prick tests, vegetation phenology remote sensing, and climate models. Research is more advanced in South Africa, creating bias within the continent (Ajikah et al., 2020; Gharbi et al., 2023a, 2023b). Additionally, methodological variation in sampling and analysis constrains direct comparisons across regions and years (Buters et al., 2018). Addressing these gaps through expanded surveillance networks, standardized methodologies, and interdisciplinary collaborations will enhance our understanding of aerospora dynamics and supporting effective management of allergen exposure and respiratory health risks across the continent. Automated spore traps have only very recently been introduced to (South) Africa (Gharbi et al., 2023a, 2023b), although the first results are not published yet.

This review synthesizes knowledge on airborne pollen and fungal spores in Africa, highlighting spatial and temporal patterns, key environmental drivers, monitoring methodologies, and public health implications. By identifying research gaps and emerging trends, we inform future aerobiology research priorities and contribute to the development of strategies, including policies for managing allergen exposure on the continent under conditions of ongoing climate change and infrastructure failure.

## Methodology

This review incorporates a structured, multi‑step process to synthesize existing research on airborne pollen and fungal spores (aerospora) across Africa. The methodology consisted of a systematic literature search, study selection, data extraction, and narrative synthesis, as detailed below.

### Literature search

Relevant scientific literature was identified through systematic searches of major bibliographic databases, including PubMed, Web of Science, Scopus, Google Scholar, and African Journals Online (AJOL). Search strategies employed combinations of keywords and Boolean operators such as: “aerobiology” OR “airborne pollen” OR “fungal spores” OR “aeroallergens” AND “Africa” OR “aerospora” AND country names (e.g. Nigeria, South Africa, Kenya, Benin, Morocco, Algeria, Tunisia, Egypt, Madagascar, Democratic Republic of the Congo, Cameroon, Gabon, Ghana, Côte d’Ivoire, Senegal). Searches were supplemented with manual review of reference lists from key articles, regional journals, and conference proceedings. Reports from continuous monitoring initiatives (e.g. South African Pollen Monitoring Network-SAPNET pollencount.co.za; Ajikah et al., 2020; Algerian Pollen repository Batna-pollen.com) were consulted. Peer‑reviewed scientific papers published in English or French were considered, regardless of publication date but with a focus on studies published between 2022 and 2026, to capture recent advances and emerging patterns. Due to the limited availability of scientific journal articles for specific countries, non-peer-reviewed publications, e.g. PhD and MSc theses, were considered.

### Inclusion and exclusion criteria

Studies were selected based on specific criteria. Inclusion criteria encompassed original field investigations reporting airborne pollen and/or fungal spore data i(e.g. Tossou et al., 2016; Tchabi et al., 2017a, 2017b; Adeonipekun, 2012; Olatunj et al., 2018; Ige & Ayeni, 2019; Ajikah et al., 2020, 2021; Doko et al., 2023); studies employing recognized aerobiological sampling methods, such as volumetric Hirst‑type traps, Burkard traps, Durham traps, or modified Tauber traps (e.g. Buters et al., 2018; Esterhuizen et al., 2023; Tossou et al., 2016); research reporting seasonal, spatial, or meteorological patterns of aerospora (e.g. Ige & Ayeni, 2019); studies linking aerospora concentrations to health, including allergic diseases or asthma (e.g. Tossou et al., 2016; Gharbi et al., 2023a, 2023b); reviews providing regional or continental syntheses of aerobiological research (e.g. Ajikah et al., 2020, 2021). Exclusion criteria included laboratory-only studies without ambient air sampling, reports lacking explicit geographic or temporal data, and publications in languages other than English or French.

### Data extraction and organization

From the selected studies, information was systematically extracted and organized into predefined categories. Geographic context included country, city, approximate latitude and longitude, and biome. Temporal coverage encompassed the sampling period and study duration. Sampling methodology included trap type, sampling frequency, and analytical techniques. Taxonomic data detailed dominant pollen taxa, such as Poaceae, Cupressaceae, and *Olea*, as well as fungal spores such as *Alternaria* and *Cladosporium*. Meteorological drivers involved reported influences of rainfall, temperature, humidity, wind speed, and seasonal winds, such as the Harmattan. Finally, health associations documented links between aerospora and allergic sensitization or respiratory symptoms, where available. Extracted data were organized by region (North, Central, West, East, Southern Africa) to support structured synthesis and regional comparisons (Table 1).

**Table 1:** Flow chart of inclusion and exclusion criteria for aerospora studies in Africa

Domain	Extracted variables/description
Geographic context	Country; city; latitude; longitude; biome
Temporal coverage	Sampling period(s); study duration
Sampling methodology	Trap type; sampling frequency; analytical/identification techniques
Taxonomic data	Dominant pollen taxa (e.g. Poaceae, Cupressaceae, *Olea*); dominant fungal spores (e.g. *Alternaria*, *Cladosporium*)
Meteorological drivers	Rainfall; temperature; relative humidity; wind speed; seasonal wind systems (e.g. Harmattan)
Health associations	Associations between aerospora exposure and allergic sensitization or respiratory symptoms (where reported)
Regional classification	North, Central, West, East, and Southern Africa

### Synthesis and comparative analysis

Qualitative and quantitative results were synthesized narratively to identify spatial patterns, key environmental drivers, and research gaps. Aerobiological data were compared across regions to highlight contrasts in dominant taxa, seasonal climate dynamics, and monitoring capacity.

### Quality assurance and limitations

Only studies with clearly described sampling protocols, taxonomic identification, and temporal context were retained. Cross‑database screening was conducted to minimize omissions. Recognized limitations include heterogeneity in methods (e.g. sedimentation vs. volumetric techniques), uneven geographic coverage (notably scarce continuous records in Central and Eastern Africa), and limited standardized reporting of meteorological covariates in older studies.

## Results

**Aerobiology research across Africa**

**Fig. 1:**
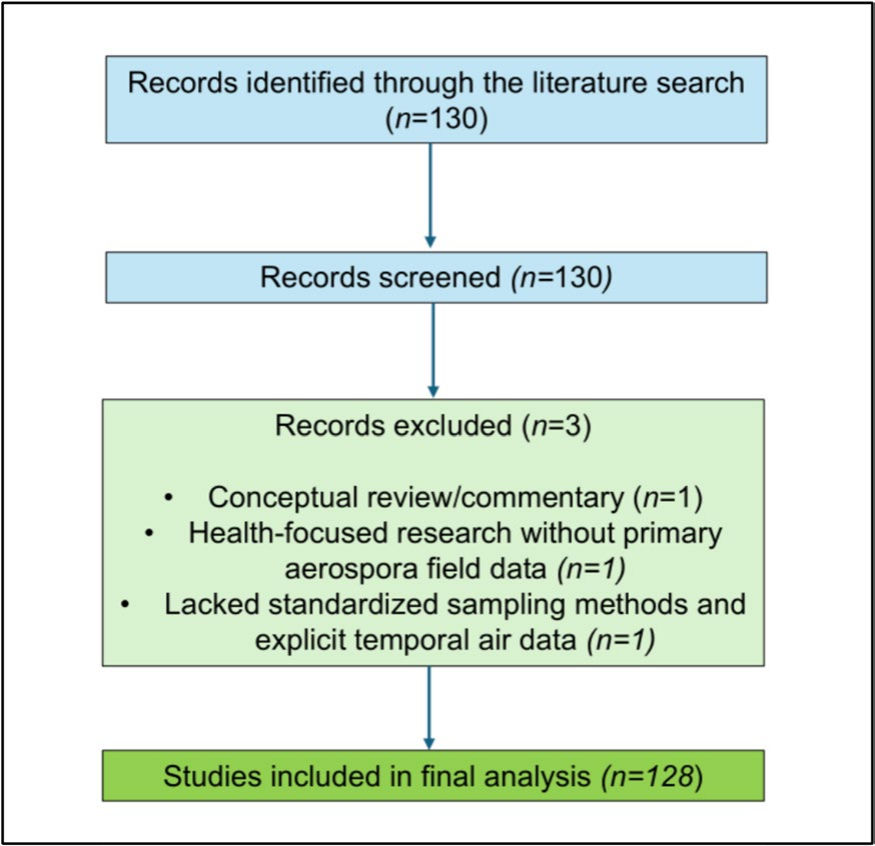
Systematic review study selection process

Using the methodology outlined in Sect. 2, this review synthesized peer-reviewed literature, theses, and monitoring reports on airborne pollen and fungal spores (aerospora) across Africa. One hundred thirty publications were identified. Three studies were excluded based on predefined criteria: one conceptual review/commentary (Gharbi et al., 2023a, 2023b), one health-focused research without primary focus on aerospora (Mbatchou et al., 2016), and one study lacking standardized sampling methods and explicit temporal air data (Fredoux & Maley, 1996). A total of 128 country-specific peer-reviewed aerobiological studies, theses, and institutional monitoring reports published between the 1970s and 2026 were considered, encompassing a wide range of sampling methodologies (volumetric and passive traps) and geographic scales (Fig. 1).

**Fig. 2:**
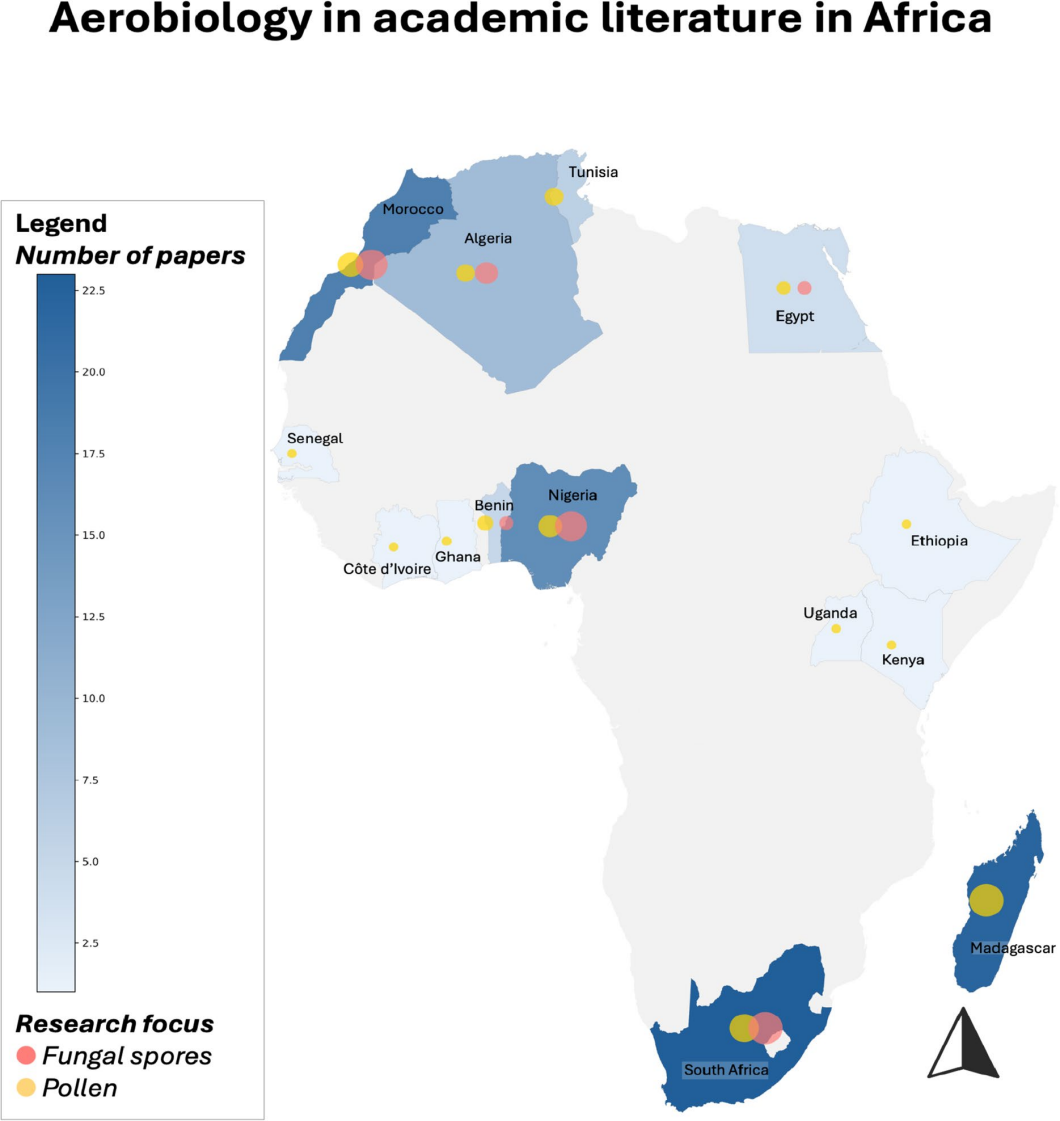
Map of aerobiology research on the African continent. The number of papers per country is presented using a blue scale, and scaled circles indicate the research focus of the papers—red for papers on fungal spores and yellow for those on pollen

Several countries were represented by only one or two studies, while Central Africa remains undocumented. The remaining publications consisted of multi-country or continental analyses. For regional clarity, the reviewed aerobiological studies were distributed across African sub-regions as follows. North Africa comprised Morocco, Algeria, Tunisia, and Egypt. West Africa included Nigeria, Benin, Ghana, Côte d’Ivoire, and Senegal. Central Africa encompassed the Democratic Republic of Congo, Gabon, Cameroon, and the Central African Republic, for which aerobiological studies were rare or entirely lacking. East Africa included Kenya, Uganda, and Ethiopia. Southern Africa comprised South Africa and Madagascar, although systematic and long-term monitoring was largely restricted to South Africa and, to a lesser extent, Madagascar. This is illustrated in Fig. 2.

### North Africa

**Fig. 3:**
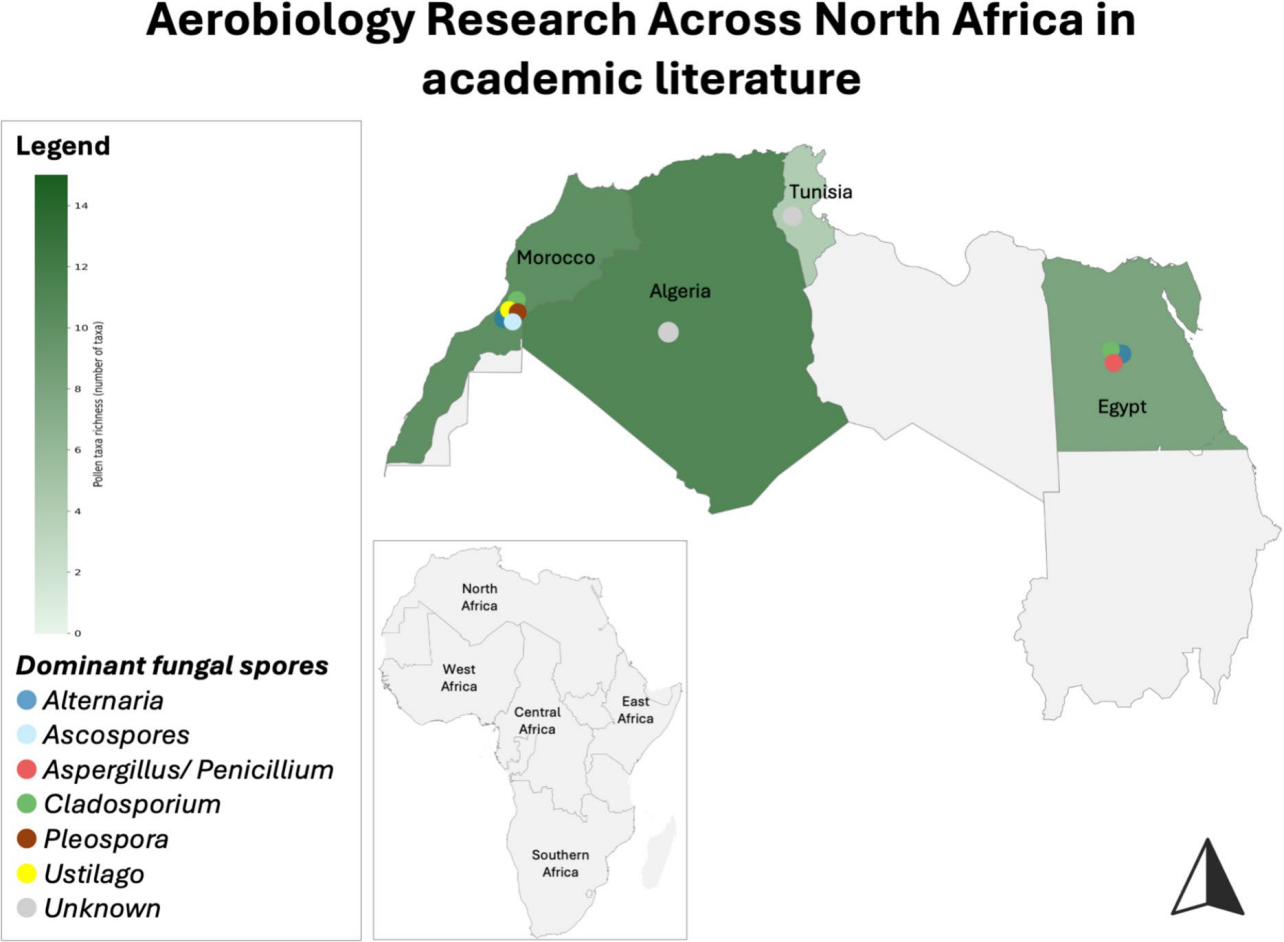
Map of aerobiology research in North Africa. The pollen taxa richness documented in the literature is indicated as a gradient of green, along with the dominant fungal spores indicated as coloured dots

Northern Africa’s coastal and montane regions experience a Mediterranean climate characterized by hot, dry summers and mild, wetter winters, which supports characteristic Mediterranean woodlands and forests dominated by evergreen oaks, pines, shrublands, and olive trees, distinct from the adjacent arid and desert biomes of the interior (Jäckle et al., 2013; Ihadadden et al., 2013). Aerobiological research in North Africa (Fig. 3) is concentrated in Morocco. While data from Algeria, Tunisia, and Egypt remains fragmented and limited to short-term surveys, Morocco has established robust, long-term monitoring networks. This success is driven by collaborations between universities, meteorological services, and European networks such as the *Red Española de Aerobiología* (REA). Aerobiological efforts in neighbouring countries are constrained by short-term project funding tied to master's or doctoral theses.

#### Morocco

Twenty-six scientific publications were identified, with 18 focusing on airborne pollen and eight addressing fungal spores. Aerobiological studies, conducted mainly in Tétouan (Northwestern Morocco), have employed volumetric Hirst‑type traps. These studies identify pollen of *Olea europaea* (olive), *Pinus* spp., Cupressaceae, Urticaceae, and Poaceae, aligned with the surrounding Mediterranean vegetation (Aboulaich et al., 2008, 2009, 2013a; Boullayali et al., 2021; El Hassani et al., 2022). *Platanus* pollen was also investigated in Tétouan, Northern Morocco (Raissouni et al., 2025), as airborne concentrations that were previously low have become more noticeable in recent years, likely related to extensive urban planting. Aboulaich et al. (2013b) documented Cannabis pollen, demonstrating seasonal variability influenced by both local sources and long-distance transport under regional meteorological conditions. Janati et al. (2017) analyzed seven years of grass (Poaceae) pollen data in Tétouan, revealing a highly variable pollen season (70–158 days) with peaks in spring and early July, and showed that daily concentrations were strongly influenced by temperature, precipitation, and relative humidity, enabling predictive modelling of airborne grass pollen. Raissouni et al. (2024a) developed multiple regression models to forecast the start and peak dates of the Poaceae pollen season, finding that pre-season meteorological factors such as minimum winter temperatures and prior autumn precipitation strongly influence the season's onset, while peak timing is shaped by early spring precipitation. Raissouni et al. (2024b) analyzed 13 years of olive pollen data, demonstrating that pre-season temperatures and precipitation significantly shape the timing, duration, and intensity of pollen seasons, with trends indicating a shortening of pollination periods. Achmakh et al. (2015) applied a thermal model based on growing degree-days to predict the start and peak of the *Olea europaea* pollen season. Achmakh et al. (2020) developed and validated multiple regression models to forecast olive crop yield and found that rainfall before flowering and during fruit growth, together with minimum summer temperatures, were identified as the main predictors. The same pollen was analyzed by Bouziane et al., (2025), applying partial least squares regression to identify the most influential thermal periods controlling the onset of pollination. Three discontinuous chilling accumulation phases were found from September to early December, along with a main heat accumulation period from mid-February to mid-April. Boullayali et al. (2023) studied chilling and heat requirements of *Olea europaea, Quercus, Pistacia*, and *Morus* in Tétouan. Their results showed that heat accumulation is the primary driver of pollination timing, while variations in chilling play a secondary role. Raissouni et al. (2025) estimated these requirements using different methods for predictive purposes. The authors concluded that the dynamic model for chilling and growing degree hours for heat provided the most robust combination for predicting the onset of *Platanus* pollen season. El Hassani et al. (2022) developed pollen calendars for the most abundant pollen types in the region; these calendars provide valuable and practical information for aerobiologists, as well as for clinical and public health professionals. However, the reliability and applicability of such tools largely depend on the sampling methodologies used to collect pollen data. In this regard, beyond studies conducted using the active method with Hirst-type samplers, Boullayali et al. (2024) evaluated a new gravimetric approach that offers several advantages, including ease of use, low cost, and no requirement for electrical power. The authors compared the results obtained with this gravimetric method to those from the volumetric method and found that both approaches were equally effective for estimating airborne *Olea* pollen concentrations. Boullayali et al. (2025) investigated long-term trends in airborne pollen across Mediterranean countries in relation to climate change, showing that temperature variations influence pollen season intensity and timing, even though the observed trends varied according to taxa, countries, and methodology. Boullayali et al. (2026) examined how the interpretation of temporal trends in the main pollen season parameters of the most allergenic taxa in the Tétouan region varied depending on the eight pollen season definitions applied. Their findings highlighted that the definition of the main pollen season remains a debated issue in aerobiology, with no single approach proving universally applicable. Furthermore, the study confirmed that no individual pollen season definition consistently outperformed the others in accurately capturing the phenological dynamics of taxa.

Fungal spores, particularly *Cladosporium* and *Alternaria,* are among the most abundant in Tétouan and exhibit pronounced seasonal maxima during warmer and moderately humid periods (Ajouray et al., 2016; Filali Ben Sidel et al., 2015, 2017). They exhibit relatively homogeneous intradiurnal distributions, with slight increases around midday, and frequently exceed allergenic threshold levels, confirming their clinical relevance as major airborne fungal allergens (Bardei et al., 2013, 2016). A recent study developed forecasting models and showed that temperature was the main factor influencing *Cladosporium* and *Alternaria* concentrations, leading to improved model performance during specific pre-peak periods (Hayoun et al., 2026). Filali Ben Sidel et al. (2015) reported that *Alternaria* spores occur year-round with peaks in spring and summer, and that temperature, humidity, and wind speed strongly influence their daily and weekly concentrations, enabling predictive modelling for allergy risk assessment. Hayoun et al., (2024), in Tétouan, demonstrated a high diversity of fungal spore types 2015–2017, with > 63 types identified, including *Cladosporium, Ustilago, Alternaria,* and *Pleospora*, and that temperature, humidity, and precipitation strongly influenced their seasonal presence and concentration. This study also developed fungal spore calendars to characterize the timing and intensity of airborne spore emissions and highlighted the potential health risks for allergic individuals exposed to high spore levels (Hayoun et al., 2024). Haskouri et al. (2016) studied ascospores such as *Leptosphaeria, Pleospora, Venturia, Diatrype, Chaetomium, Sporormiella*, and *Ascobolus* using canonical correspondence analysis and Spearman correlation. They found that meteorological variables explained 27.4% of ascospore variability, mainly driven by maximum temperature (10.3%). In the year-specific analysis, 2012 showed the highest explained variance (28.6%), dominated by minimum relative humidity (8%). Most ascospores were positively correlated with humidity and rainfall, whereas Chaetomium showed opposite responses, being favoured by temperature.

A multicenter clinical study of 640 patients across Morocco found that olive pollen is the leading cause of pollen sensitization (19.8%), followed by grass pollen (10.9%), and that sensitization prevalence was highest in regions with abundant olive trees (Yazidi et al., 2001). In parallel, a clinical study conducted in Casablanca reported a substantial prevalence of cypress pollen sensitization: among 154 patients consulting for the first time for asthma and/or rhinitis and/or conjunctivitis, 32 patients (20,78%) showed positive skin prick test reactions to cypress pollen (Afif et al., 2006). In addition, a recently published study by Bouaissa (2026) reviews and critically evaluates clinical and aerobiological data published in Morocco from 2000 through 2025, with a focus on pollen allergen sensitization patterns, aeroallergen exposure assessments, and associated clinical outcomes. Key pollen taxa, methods of aeroallergen monitoring, prevalence trends of sensitization in different demographic groups, and the interplay between environmental drivers and allergic disease expression are examined. The synthesis highlights both consistent patterns of sensitization to common pollen families such as olive tree (*Olea europaea*) and grasses (Poaceae), *Cupressus* (Cupressaceae), *Parietaria, Corylus,* and *Acacia*, defined as the top pollen allergens in Morocco, and significant gaps in standardized pollen monitoring and clinical reporting (Bouaissa, 2026).

#### Algeria and Tunisia

In Tunisia and Northern Algeria, aerobiological evidence remains limited and geographically fragmented, as most available information still derives from short-term surveys and localized monitoring rather than long, harmonized time series. In Algeria, aerobiological research comprises ten published studies, including early gravimetric surveys, several short-term volumetric studies, and a limited number of clinical investigations, with most data originating from Annaba and Batna. In contrast, in Tunisia, the available literature includes six published aerobiological studies, primarily short-term or multi-site surveys using Hirst-type samplers, complemented by a small number of clinical allergy studies.

***Algeria***

Aerobiological research in Algeria has progressed from early passive and gravimetric surveys to standardized volumetric monitoring, although evidence remains spatially fragmented and largely concentrated in northern and eastern regions. Early aeropalynological studies were conducted in north-western Algeria, especially Oran Province, using the Cour method between 1977 and 1979 (Cambon, 1981), while parallel investigations in the Algiers region used the Durham method (Korteby-Becila, 1987). More advanced Hirst-type monitoring was later performed in Algiers from 2001 to 2003 (Gharnaout, 2007; Ghernaout et al., 2009), and additional Cour filter-based monitoring was carried out at Es-Senia airport, Oran, between April 2004 and April 2006 (Kiared et al., 2016). Together, these studies indicate that Algeria’s Mediterranean, Saharan-influenced and semi-arid gradients generate seasonally structured aeroallergen patterns dominated by weed and herbaceous pollen, such as Chenopodiaceae–Amaranthaceae, *Plantago*, Urticaceae, Brassicaceae and Apiaceae, together with arboreal pollen including *Olea*, Cupressaceae, *Casuarina, Quercus, Pinus* and *Eucalyptus* (Cambon, 1981; Gharnaout, 2007; Ghernaout et al., 2009; Kiared et al., 2016).

Additional surveys from north-eastern Algeria and Algiers confirm the regional relevance of these taxa. In Annaba–El-Hadjar, a one-year gravimetric survey identified 50 airborne pollen taxa and a marked late-winter to early-spring maximum (Necib & Boughediri, 2016), whereas the Guelma survey recorded 3,348 pollen grains from 33 families, with a predominance of anemophilous taxa and 65% of families considered allergenic, mainly in March–April (Chahat et al., 2017). For Algiers, the Hirst-type reference dataset remains particularly important, showing Cupressaceae as the main contributor (26.52% in Algiers Centre, 44.24% in Algiers Plateau), with a late-winter peak, together with substantial Urticaceae and *Olea europaea* contributions (Gharnaout, 2007; Ghernaout et al., 2009). These results also underline the need for North Africa-specific exposure baselines, since some major European aeroallergens, such as birch and ragweed, are absent or poorly represented in the Algerian spectra (Ghernaout et al., 2009).

Amroune et al. (2026) strengthen the Algerian evidence base by providing standardized Hirst-type monitoring data from Batna and its surrounding areas, Tazoult and Hamla, in the semi-arid Mediterranean zone of eastern Algeria. Conducted over a complete pollen year from 1 November 2022 to 31 October 2023 using a Lanzoni VPPS 2000 sampler, the study recorded 31,853 pollen grains/m^3^ distributed among 34 pollen types and 28 families (Amroune et al., 2026). The pollen spectrum was strongly dominated by trees and shrubs (85.40% of the annual pollen index), followed by herbaceous taxa (12.70%) and Poaceae (1.60%) (Amroune et al., 2026). Four taxa accounted for 83.23% of the annual pollen load: Cupressaceae (66.28%), *Quercus* sp. (9.77%), Chenopodiaceae–Amaranthaceae (3.80%) and *Olea europaea* (3.38%) (Amroune et al., 2026). The temporal profile was also highly concentrated, with 88.41% of the annual pollen load occurring between February and June, and March alone representing 52.28% of the annual index, mainly due to an intense Cupressaceae peak (Amroune et al., 2026).

From a clinical and public-health perspective, the Algerian evidence now highlights three main periods of elevated allergenic exposure: a winter period dominated by Cupressaceae, a spring to early-summer period involving *Quercus, Olea, Fraxinus, Plantago*, Urticaceae and Poaceae, and a late summer–autumn period dominated by Chenopodiaceae–Amaranthaceae, *Casuarina* and *Artemisia* (Amroune et al., 2026). These environmental observations are supported by clinical data from Algiers, where a retrospective series of 77 pollen-sensitized patients reported rhinitis (83%) and asthma (52%) as the most frequent manifestations, with sensitization dominated by grasses (62%), followed by tree pollen (32%) and herbaceous pollen (27%); olive was the leading tree pollen and *Parietaria* the most sensitizing herbaceous pollen (Sayah et al., 2021). Additional clinical evidence from central Algeria suggests regional variability, with lower sensitization rates to olive and cypress (Bounil et al., 2022). Overall, Algeria should no longer be presented as simply data-poor, but rather as an emerging and regionally uneven aerobiological setting where coastal, semi-arid and inland exposure profiles must be compared through expanded, multi-year Hirst-type monitoring, clinical integration and public-facing risk communication, including tools such as the Batna-Pollen platform (Batna Pollen, n.d.).

***Tunisia***

Aerobiological research in Tunisia includes six studies, documenting airborne pollen dynamics for agricultural forecasting, regional pollen spectra, and clinical sensitization patterns. In Tunisia, aerobiological studies were focused on modelling the olive-crop forecast using pollen data from main olive-producing areas and the principal cultivars, i.e. Chetoui and Chemlali, using data collected by a Cour trap. A pioneer comparative airborne pollen surveys across Spain, Italy and including Tunisia from 1993 to 2012, using Hirst‑type traps, was conducted to determine models that study the parameters influencing olive fruit production in selected countries (Oteros et al., 2014). For the case of Tunisia, water availability in spring (rainfall minus crop evapotranspiration from 1 October to 30 April) and March rainfall explained the significant *Olea* fruit tree production. A similar study conducted by Ben Diab et al., (2017) using phenoclimatic variables showed that amount, timing, and distribution of rainfall (except during blooming) had a positive impact on final olive harvests. In another context, Orlandi et al. (2014) conducted a quantitative study to downscale the differences from two of the main air traps used on aerobiology, the Hirst and Cour air samplers, at three Tunisian sampling points (Oteros et al. 2014), from 2009 to 2011. Later on, a study aiming to determine the distribution of the different spring flowering species from the North to the South of Tunisia and to establish the pollen spectrum of three regions Mornag (North), Menzel M’hiri (Centre) and Chaal (South) in 2017, showed consistently abundant alongside Cupressaceae, Poaceae, and Amaranthaceae, with variation in total pollen indices and taxa distribution among regions (Hadj Hamda et al., 2019). Regarding applied aerobiology to respiratory health, clinical data similarly point to a substantial contribution of herbaceous pollen, with herbaceous sensitization reported as predominant (54.4%) alongside high sensitization to tree and grass pollen (Yangui et al., 2018). In addition, a longitudinal study by Yangui et al., (2015) comparing two consecutive 10-year periods (1995–2004 (P1) and 2005–2014 (P2) showed a significant increase in overall pollen sensitization from P1 to P2 (38.6% vs 49.7%, p < 10⁻⁶), mainly driven by higher sensitization to tree (12.7% vs 18.5%) and grass pollen (14.6% vs 18.2%). Notably, sensitization to cypress pollen increased markedly (5.6% vs 12%).

#### Egypt

In Egypt, aerobiological research has been limited and largely exploratory, with four cited studies with almost three studies that were identified focusing on urban environments along the Nile Valley and Delta rather than systematic national monitoring. A one‑year volumetric pollen trap study in Rosetta (Nile Delta) documented airborne pollen assemblages from Poaceae (grasses), Arecaceae (palms), Chenopodiaceae–Amaranthaceae, *Casuarina* (Australian neophytic tree), Cupressaceae, Urticaceae, *Pinus*, and Myrtaceae, with the highest pollen counts from February to May, yielding a preliminary, regional pollen calendar (Taia et al., 2019). Aerobiological surveys in Alexandria using Hirst‑type traps also reported annual airborne pollen presence alongside diverse fungal spores, with Chenopodiaceae/Amaranthaceae, *Casuarina*, Arecaceae, Pinus, and Urticaceae among the dominant taxa, and fungal spores—principally *Cladosporium, Alternaria*, and *Aspergillus/Penicillium* types—exceeding pollen counts in total spore index (Bassiouni & Taia, 2021). These pollen spectra reflect the influence of urban vegetation, irrigated agriculture, and widespread grass, date palm, and weed species on airborne aerospora in lower‑latitude Mediterranean and semi‑arid settings. Clinical studies, while not direct aerobiological monitoring, support the allergic relevance of airborne pollen in Egyptian cities. Among respiratory allergy patients in Cairo and surrounding areas, grass pollen (e.g. timothy grass (*Phleum pratense*) and maize (*Zea mays*) are the most prevalent sensitizers detected by allergy skin prick testing, highlighting key local aeroallergens where pollen data are sparse (Refaat et al., 2021; Mohammed et al., 2022). In contrast to pollen, airborne fungal spore dynamics remain poorly characterized outside a few localized investigations, representing a significant knowledge gap given Egypt’s large urban and agricultural populations and the high burden of respiratory allergies.

The available aerobiological and clinical evidence underlines the need for systematic, long‑term monitoring networks that integrate volumetric pollen/spore trapping, meteorological data, and public health surveillance to better assess allergen exposure and the effects of irrigation, temperature regimes, and urbanization on aerospora dynamics across North Africa. Overall, North Africa represents a critical but underrepresented region in continental aerobiological research. Expanding long‑term monitoring, standardizing sampling methodologies, and linking aerobiological data with clinical allergy surveillance are essential steps towards improving understanding of aeroallergen patterns and mitigating associated health risks in the region.

### Central Africa

**Fig. 4:**
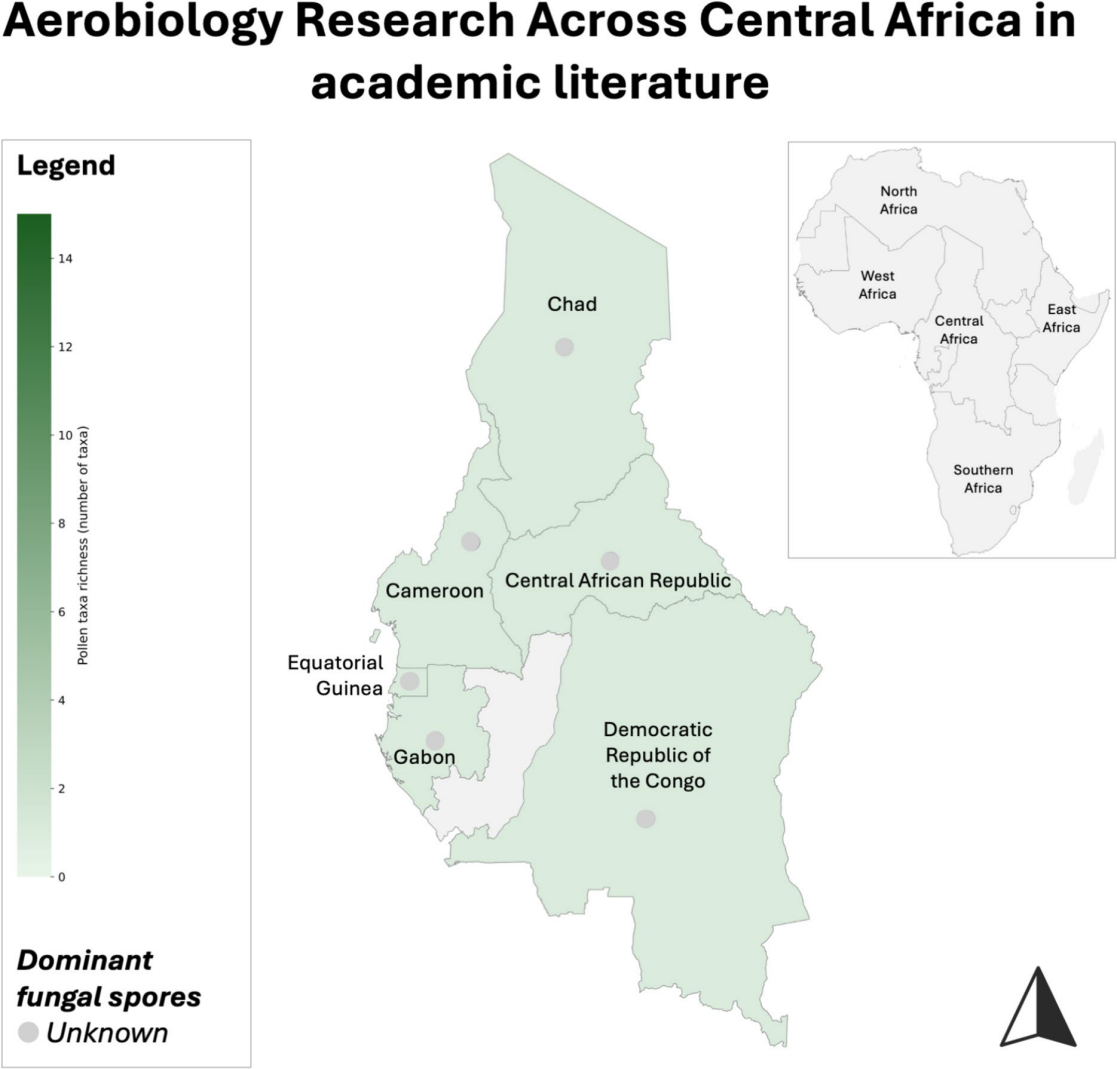
Map of aerobiology research in Central Africa. The pollen taxa richness documented in the literature is indicated as a gradient of green, along with the dominant fungal spores indicated as coloured dots

Aerobiological research in Central Africa is extremely limited (Fig. 4), with only three cited studies documenting airborne pollen and fungal spores, highlighting the lack of long-term monitoring and underscoring the urgent need for systematic aerobiological and aeromycological investigations in this highly biodiverse region (Adeonipekun et al., 2020; Garba et al., 2022). Countries such as the Democratic Republic of Congo, Cameroon, and Gabon—despite encompassing some of the largest and most biodiverse tropical rainforest ecosystems globally—remain largely absent from continental aerobiological datasets. Consequently, the current understanding of airborne pollen and fungal spore dynamics in this region remains highly fragmentary.

Available information is derived from brief aerobiological surveys or palynological and aeromycological studies not specifically designed for continuous airborne monitoring. These limited observations suggest that tropical rainforest vegetation contributes to exceptionally high pollen and spore diversity, reflecting the richness of arboreal taxa, understory plants, lianas, and epiphytic flora characteristic of Central African forests (Lézine et al., 2019). In addition to pollen from diverse woody and herbaceous taxa, fungal spores are presumed to be abundant due to persistently high humidity, warm temperatures, and dense organic matter; however, their taxonomic composition, seasonal variability, and allergenic relevance remain poorly documented in the absence of targeted aeromycological investigations (Garba et al., 2022).

Climatic conditions in Central Africa—characterized by tropical conditions with high annual rainfall, weak temperature seasonality, and frequent convective precipitation—produce aerobiological patterns that differ markedly from those observed in Mediterranean, savanna, or semi-arid regions of Africa. Despite this, no long-term aerobiological records currently exist to assess seasonal trends, interannual variability, or the influence of climate variability and land-use change on aerospora concentrations in the region.

The near absence of standardized aerobiological monitoring infrastructure, such as volumetric pollen traps and harmonized identification protocols, represents a major research gap that has been repeatedly highlighted in continental-scale aerobiological assessments (Grewling et al., 2023). Establishing baseline monitoring stations in Central African urban and peri-urban centres, combined with meteorological observations and public health surveillance, is essential for advancing understanding of aeroallergen exposure in this region. Addressing this gap is particularly urgent given rapid urbanization, deforestation, and the expected sensitivity of tropical aerospora dynamics to ongoing climate change.

### West Africa

**Fig. 5:**
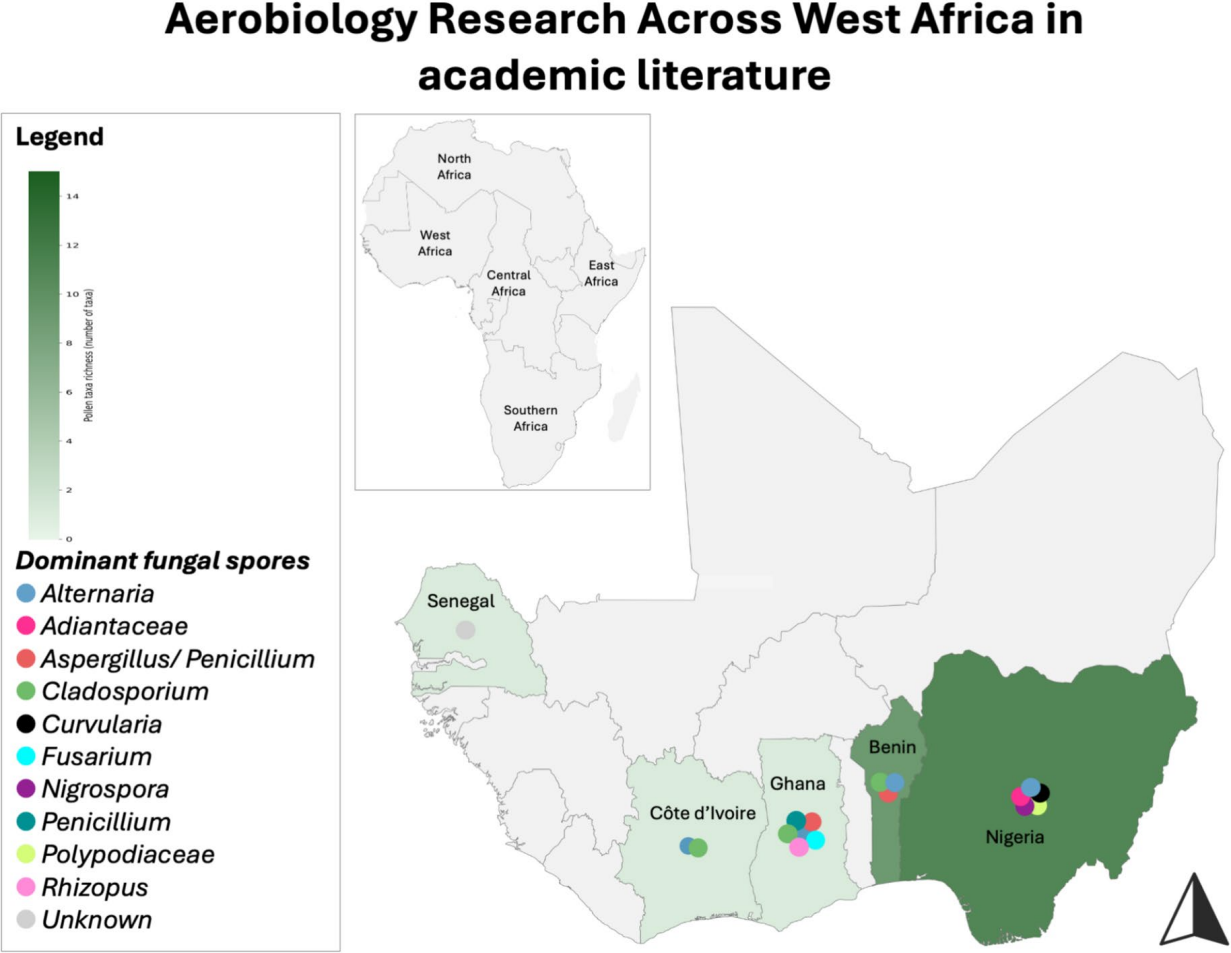
Map of aerobiology research in West Africa. The pollen taxa richness documented in the literature is indicated as a gradient of green, along with the dominant fungal spores indicated as coloured dots

West Africa (Fig. 5) exhibits a pronounced tropical climatic gradient from the humid Atlantic littoral in the south to the semi-arid Sahel in the north, shaped largely by the seasonal north–south migration of the West African Monsoon system (Nicholson, 2013). The southernmost zones receive high rainfall (> 1600 mm yr⁻^1^) with long wet seasons, supporting tropical rainforest and moist deciduous forest ecosystems, including closed-canopy forests and coastal mangroves of the Guineo–Congolian belt. These forests are dominated by evergreen and semi-deciduous broadleaf trees such as *Khaya* spp., *Entandrophragma *spp., *Milicia excelsa*, and T*riplochiton scleroxylon*, and have been the subject of detailed studies emphasizing gradients of woody cover and disturbance processes in relation to climate and land use (Akoègninou, 1984; Akoègninou et al. 2006; Adomou, 2005).

West Africa represents one of the better-documented regions of the continent in terms of aerobiological research due to sustained studies conducted in Nigeria that has a rich palynological tradition, pioneered by late Prof. Margaret Adebisi Sowunmi, professor of palynology and environmental archaeology at the University of Ibadan (Orijemi et al., 2025). West Africa encompasses a broad climatic gradient from humid tropical rainforests in the south through savanna and woodland belts to semi-arid Sahel and arid zones in the north, supporting diverse vegetation types (moist broadleaf forests, forest-savanna mosaics, and grass-dominated savannas) that reflect seasonal rainfall patterns and underpin high regional biodiversity and ecosystem services (IUCN/PACO 2016). This diversity in natural landscapes results in pronounced spatial and seasonal variability in airborne pollen and fungal spores (Adeonipekun & John, 2011; Ezike et al., 2016; Tossou et al., 2016; Ajikah et al., 2021; Akasoro et al., 2026).

#### Nigeria

Numerous studies documented airborne pollen and fungal spores, their seasonal and spatial variation, meteorological and vegetation influences, and methodological aspects, providing a foundation for public health and continental-scale research. Nigeria has many different geographic zones (i.e. savanna, rainforest) and climatic gradients (Ajikah et al., 2021). Many different studies on aerobiology have been conducted over long and short-term time scales in various types of environments (urban, coastal, rainforest and savanna), creating a strong body of knowledge about atmospheric bioaerosols. Urban studies, i.e. in Garki, Abuja, Lagos, show that aerosol concentrations are predominantly governed by seasonal rainfall regimes and wind systems associated with the Harmattan dry season (Adeonipekun et al., 2020; Ezike et al., 2016). Fungal spores are found at high concentrations in the rainy season, which favours fungal growth. Conversely, pollen concentrations are higher during the dry season, especially during the Harmattan, as the northeasterly winds allow for long-distance transport, atmospheric persistence and resuspension of pollen (Ezike et al., 2016).

In North-Central Nigeria, studies examining the influence of meteorological variables (i.e. wind speed, humidity, temperature, and rainfall) support the previously mentioned findings regarding the environmental factors regulating pollen and fungal spores in the airstream. Multiple studies have reported that fungal spores are at peak concentrations during the wet season and pollen grains are at peak concentrations during the dry season, highlighting the strong association of seasonal climatic patterns with the distribution of aerobiological particles (Adeonipekun et al., 2020; Garba et al., 2022). Essien et al. (2024) examined the diversity, abundance, and health implications of airborne pollen and fungal spores in a semi-arid region of northern Nigeria and recovered over 29,000 palynomorphs, comprising 77 pollen types and multiple fungal taxa. Dominant pollen types included Poaceae and other known allergenic plants, while fungal spores such as *Nigrospora*, *Curvularia,* and *Alternaria* were also observed. The study identified clear seasonal peaks, with higher concentrations during dry and transitional periods. Meteorological factors, particularly wind speed and temperature, played a significant role in dispersal patterns, with evidence suggesting long-distance transport of pollen. Their research provides baseline aerobiological data for the region and underscores the need for regional pollen calendars. It contributes to understanding how climate variability influences aeroallergen distribution in Africa. The trends identified in Nigeria mirror the trends seen in other tropical regions, wherein the release of fungal spores is closely associated with the availability of moisture, while the dispersal of pollen is driven by wind and vegetation phenology. Along with being important for understanding aerobiological dynamics, studies on Nigerian aeropalynology at the national level highlight the need for public health interventions to address asthma and allergic rhinitis associated with pollen and fungal spores. As allergy and asthma rates are increasing in urban populations, further research is warranted to create a better understanding of the impact of meteorological variables on aeroallergens in densely populated coastal megacities, such as Lagos (Adeonipekun & John, 2011; Garba et al., 2022).

A need for integrated frameworks exists whereby information from aerobiological monitoring studies, empirical meteorological observations, and clinical allergy information can be incorporated to inform compliance with existing allergen forecasting, risk assessment, and intervention strategies (Ezikanyi et al., 2018). Several factors contribute to the prevalence of airline pollen in Nigeria, which include the taxonomic composition of vegetation, climatic variability, and the geographic/topographic characteristics of each study site. Therefore, the applicability of the findings from aeropalynological studies is dependent on three methodological pillars: the efficient capture of pollen via the placement of adequate pollen traps, ensuring a rigorous method of counting and identifying pollen to minimize analytical error, and effectively utilizing information from pollen data for public health, environmental, and allergenic uses. Although important progress has been made, Nigeria has yet to realize the full potential of aerobiological research with respect to how to translate the data into applicable environmental and clinical policies. Methodological limitations also exist in relation to the collection of pollen samples by traditional sampling methods. Historical studies on the efficacy of the Tauber pollen sampler, a passive system consisting of a buried container with a preservative solution that captures pollen washed in by rain and gravity, providing pollen accumulation rates (PAR) which has been the primary collection device used in Nigeria to date, reported issues with capturing small pollen grains (Peck et al., 1972), and with reliability during the wet season, as the presence of raindrops can influence the capture of pollen from the ground (Krzywinski, 1977). The increased frequency and intensity of rainfall events that are typical of tropical climates can also influence methodological limitations. Despite these limitations, many aerobiological studies have been completed in Southwestern Nigeria using the Tauber sampler, significantly contributing to the collective body of knowledge regarding regional aerobiology. For instance, Adekanmbi et al. (2019) investigated the aeroflora and potential for allergic reactions by dominant airborne pollen types in select locations within Southwestern Nigeria. They further showed that, following exposure to pollen proteins from *Tridax procumbens* and *Alchornea cordifolia*, *Mus musculus* exhibited statistically significant changes in their haematologic and serologic results. Mice that were exposed to *Alchornea cordifolia* pollen developed alopecia, suggesting that the taxon has allergenic potential. Ibigbami and Adeonipekun (2020) studied airborne pollen grains and fungal spores in two communities in Lagos State, Nigeria, Ipaja and Ikeja, to examine their relationship with allergy cases and weather conditions. They sought to identify types of allergenic pollen associated with respiratory allergies, including asthma and hay fever. Weekly air samples were collected for six months, resulting in 2,868 pollen grains and spores from 21 plant families. Ipaja had significantly higher pollen concentrations than Ikeja due to its richer vegetation. Dominant allergenic pollen types included Poaceae, Amaranthaceae, and *Alchornea cordifolia*. Two peaks in pollen levels were noted in April and June, aligning with increased allergy symptoms. Their study also found that temperature and rainfall positively correlated with pollen abundance, while humidity and wind speed generally reduced it. A significant correlation was observed between pollen levels and cold/catarrh cases in Ikeja. The researchers concluded that both airborne pollen and fungal spores significantly contribute to allergy risks in Lagos, recommending ongoing aerobiological monitoring and the development of a pollen calendar in Nigeria.

Adeniyi et al. (2017) investigated the temporal and spatial distribution of airborne Poaceae pollen across five locations in Lagos, with emphasis on its aerobiological and public health relevance. Using systematic sampling, the research quantified pollen concentrations and examined the influence of meteorological parameters. Results showed clear seasonal variation, with pollen counts peaking at different times depending on location, while the lowest concentrations occurred around June and July. Relative humidity was identified as a major factor influencing pollen abundance, often showing an inverse relationship with pollen counts. The study also demonstrated that urban microenvironments affect pollen dispersion patterns. Importantly, Poaceae pollen was confirmed as a dominant and consistent component of the aeroplankton, posing a significant allergy risk. The findings highlight periods of high exposure and suggest safer windows for allergy-prone individuals. Moreover, Adeniyi et al. (2018a, 2018b) focused on identifying allergenic proteins present in dominant airborne pollen species in Nigeria and evaluating their role in allergic reactions among hypersensitive individuals. The study examined four common pollen-producing plants: *Alchornea cordifolia, Amaranthus hybridus*, *Casuarina equisetifolia,* and *Terminalia catappa*. Pollen grains were harvested from plant anthers, and their proteins were extracted, quantified, separated using SDS-PAGE, and tested for allergenicity through Western blot analysis using blood sera from allergy patients in Lagos. Results showed that *Amaranthus hybridus* had the highest protein content, while *Casuarina equisetifolia* had the lowest. However, allergenic strength differed from protein quantity. The most allergenic pollen protein was the 58 kDa protein in *Alchornea cordifolia,* which reacted positively with 84% of the allergy patients tested. The study identified several allergenic proteins, including Profilin and PR-10, that were linked to known allergens from other plant species. These proteins were newly registered in international proteomic databases because no previous Nigerian records existed. The researchers concluded that these pollen allergens are significant contributors to allergy risks in Nigeria and could support the future development of local immunotherapy treatments and allergy vaccines.

Kemabonta et al. (2018) conducted a study focusing on a less explored aspect of aerobiology: airborne insect fragments. They assessed the occurrence, diversity, and environmental drivers of these fragments. The research identified various insect body parts, particularly legs, wings, and hairs, as significant components of the aeroplankton. Their quantitative analysis revealed that insect fragments were abundant and, at times, comparable in frequency to pollen and spores. A statistical evaluation showed strong correlations between the abundance of insect particles and climatic variables such as rainfall, temperature, and relative humidity. Seasonal trends indicated that concentrations were higher during periods of increased biological activity and environmental disturbance. The study emphasized that insect fragments can act as aeroallergens, potentially contributing to respiratory irritation and allergic reactions. Furthermore, it expanded the scope of aerobiological studies in Africa by demonstrating that bioaerosols include more than just pollen and fungal spores. The findings underscore the need to incorporate insect particulates into allergy risk assessments and monitoring frameworks. A study from the university campus of Ibadan further inland recovered dominant pollen from rainforest, secondary forest, savanna and freshwater habitats, underlining that the Harmattan season poses the greatest health risk due to strong allergenic pollen loads (Akasoro et al., 2026).

While Okwong et al. (2019) explored a second methodology by demonstrating that spider webs could be used as natural spore traps in urban landscapes (i.e. Lagos), the data were limited by biases. Results from their study indicated that high pollen volumes were observed March–August. Low concentrations were detected November–January, consistent with the effects of seasonal vegetation and climate on pollen production. Ajikah et al. (2017) conducted an aerobiological study in Lagos and documented the most significant pollen load in May and the lowest number of pollen loads in June, including a strong correlation with climatic parameters. Earlier investigations conducted by Ajikah et al., (2015) at the University of Lagos located the presence of fungal spores (Polypodiaceae and Adiantaceae) along with pollen from Poaceae, Cyperaceae, Sapotaceae, Combretaceae and Euphorbiaceae as significant contributors to the aerobiological community of the university's campus. Likewise, Adeniyi et al. (2014) documented yearly distribution patterns of pollen in Southwestern Nigeria, highlighting high-risk periods and linking pollen presence with both allergy and meteorological data. Adeonipekun (2012) provided additional support for the argument in review by showing evidence of long-distance pollen transport in Ayetoro-Itele, Ota. The findings of his study included the observation that the highest number of samples collected were of bald cypress, Amaranthaceae, Poaceae, and also a much lower number of samples of savanna-type vegetation were noted, which contradicted earlier research findings. The discrepancy was attributed to the timing and intensity of the Harmattan wind and, therefore, the ability for long-distance transport of savanna-type pollen into wooded areas, which is commonly observed in northern regions of Africa. Overall, cumulative research in Nigeria on aerobiological inquiries has documented consistent patterns in yearly variation of aerobiological data and emphasized the importance of understanding the interplay between local vegetation and climate on the presence of pollen and spores in the atmosphere. Research performed in the southeast, southwest, and north central regions of Nigeria (Agwu & Osibe, 1992; Ezike et al., 2016; Njokuocha, 2006; Njokuocha et al., 2017) have demonstrated how changes in the timing and volume of moisture availability through seasonal cycles ultimately affect the volume of pollen production between two different ecological areas. Furthermore, environmental monitoring studies have demonstrated how aerobiological monitoring can provide valuable information regarding vegetation movement and anthropogenic displacement.

Nigeria represents a strong, sustainable centre for research in aerobiology within Africa and provides a considerable amount of empirical data regarding both the dynamic nature of pollen and spores as well as related health and methodological issues. Therefore, the information collected in Nigeria can be used as a foundation for establishing aerobiological research programmes at the continental level, particularly in terms of how the influence of tropical climates affects aerobiological research and the integration of aerobiological data into public health frameworks.

#### Benin

Aerobiological research in Benin has begun to characterize the composition and seasonal variation of airborne pollen, linking these patterns with local climate and vegetation. Five aerobiological studies have documented airborne pollen and fungal spores. Earlier gravimetric sampling on the Abomey‑Calavi, which started in 2013 using Durham samplers, recorded ~ 5,053 pollen grains across 21 families, with peaks during the dry season likely influenced by wind and evapotranspiration, highlighting climatic modulation of airborne pollen in the region (Tossou et al., 2016). Afterwards, a Hirst‑type volumetric study in Abomey‑Calavi, conducted from 2015 to 2017, documented daily means of ~ 35 pollen grains/m^3^ with dominant taxa including *Acacia auriculiformis*, Poaceae, *Combretum indicum*, and *Elaeis guineensis*, showing that pollen concentrations correlated positively with temperature and wind speed but negatively with rainfall (Tchabi et al., 2017a). In parallel, in 2015, an aerobiological survey was conducted during the rainy season study (Tchabi et al., 2017b). Daily averages of 35 pollen grains/m^3^ were found using Hirst sampling. Dominant taxa were *Acacia auriculiformis*, Poaceae, *Combretum indicum*, and *Elaeis guineensis*. Correlations showed positive associations with temperature and wind speed, and a negative association with rainfall, suggesting meteorological modulation of dispersal (Tchabi et al., 2017a, 2017b). About the spore content, air sampling of the atmosphere of Abomey-Calavi was also analyzed during the same period in relation to clinical data on allergic diseases from the hospital of the same municipality. A Hirst-type sampler was used to collect these particles. A total of 134 spores/m^3^ of air per day was recorded from April 2015 to March 2017. A seasonal generic variation of airborne spores was observed. Thus, during the dry season, the most dominant taxa were *Aspergillus*, *Cladosporium*, and *Alternaria*, whereas during the rainy season, *Aspergillus* and *Cladosporium* spores predominated. The data collected on spores during the study are of great importance to help allergists prevent, diagnose, and raise public awareness about allergic diseases (Tossou et al., 2016). Following that, a year‑long study (Nov 2019–Nov 2020) in Cotonou counted nearly 19,921 pollen grains across ornamental and non-ornamental taxa. Ornamental plant pollen constituted 28% of this total, with non‑ornamentals dominant (72%). Species frequently observed included *Pithecellobium dulce*, *Terminalia mantaly*, *Euphorbia milii*, *Celosia cristata*, as well as Australian neophytic trees such as *Casuarina equisetifolia* and *Acacia auriculiformis* (Doko et al., 2023).

#### Other West African countries

Other regions of West Africa are recognized for their weak aerobiological research capacity. Outside of Nigeria and the Republic of Benin, there are large gaps in the systematic/long-term monitoring of airborne pollen and fungal spores. In West Africa (including Ghana, Côte d'Ivoire and Senegal), aerobiological research is limited, as the observation summarized by D’Amato et al., (2020) points out significant knowledge gaps and the need for sustained monitoring efforts. Furthermore, their review highlighted the dominance of grass pollen and fungal spores in urban and peri-urban environments and underscored the urgent need for long-term, multi-site monitoring to understand seasonal dynamics and public health impacts.

In Ghana, few studies have been carried out, notably Yafetto & Adator (2018) who investigated the occurrence, diversity, and concentration of airborne fungal spores in indoor and outdoor environments within selected buildings at the University of Cape Coast, Ghana. Their study aimed to assess fungal contamination levels and determine potential health risks associated with exposure to airborne fungi in educational settings. Air samples were collected from lecture halls, offices, laboratories, libraries, and surrounding outdoor areas using passive air sampling techniques. The fungal isolates were cultured, identified morphologically, and analyzed statistically to compare indoor and outdoor fungal distribution. The results showed that fungal spores were more abundant outdoors than indoors; although some indoor locations also exhibited high fungal loads due to poor ventilation and occupancy activity (i.e. dusting of furniture, vacuum cleaning). Dominant fungal genera identified included *Aspergillus*, *Penicillium*, *Cladosporium*, *Fusarium*, and *Rhizopus*. Factors such as humidity, temperature, dust accumulation, and overcrowding significantly influenced fungal proliferation. In some cases, fungal concentrations exceeded acceptable indoor air quality standards, highlighting potential health risks for building occupants, including respiratory allergies, asthma, skin irritation, and other hypersensitivity reactions.

Larrey et al. (2020) examined airborne bacteria and fungi in the indoor environments of a teaching hospital in Ghana to assess indoor air quality and health risks. Air samples were collected from various clinical areas, like wards and laboratories, using passive settle plate methods to isolate and identify microorganisms. The results indicated significant variation in bacterial and fungal loads, with some wards exceeding internationally recommended indoor air quality limits. The dominant bacterial taxa included *Staphylococcus aureus* and *Bacillus species,* while common fungal isolates were *Aspergillu*s and *Penicillium*. These microorganisms are known to cause respiratory infections and allergic reactions, particularly in immunocompromised individuals. Poor ventilation and inadequate sanitation contributed to higher microbial contamination. The presence of airborne fungal spores was associated with an increased risk of asthma and nosocomial infections, underscoring the need for continuous monitoring, improved ventilation systems, and strengthened infection control measures in healthcare facilities. In addition, these fungal and bacterial taxa include some of the most abundant aeroallergens worldwide and are recognized as important triggers of allergic respiratory diseases (e.g. asthma and allergic rhinitis) in susceptible individuals (D'Amato et al., 2020).

The scarcity of long-term aerobiological monitoring in Ghana, particularly the lack of continuous multi-year data collected using volumetric trapping methods, has significantly hindered the development of reliable pollen calendars for Ghana and limits robust assessments of intra- and inter-annual aerospora dynamics. In contrast, where time-series data are available, they consistently demonstrate that seasonal peaks in airborne pollen and fungal spores are closely associated with West African meteorological patterns, particularly the timing of seasonal moisture influx. During the late dry and early wet transition periods, grass-dominated vegetation reaches reproductive maturity, resulting in elevated concentrations of grass pollen in the atmosphere. Conversely, during the wetter and more humid months, increased moisture availability promotes fungal growth, reproduction, and sporulation, leading to higher concentrations of airborne fungal spores.

These observations are consistent with findings from localized aerobiological and environmental microbiology studies in Ghana, such as those by Yafetto & Adator (2018) and Larrey et al., (2020), which demonstrate that environmental conditions—including humidity, temperature, ventilation, and human activity—strongly influence airborne fungal loads in both indoor and outdoor environments. The taxa identified in these studies include major aeroallergens such as *Aspergillus*, *Penicillium*, and *Cladosporium*, reinforcing their relevance to respiratory allergy risk in tropical urban settings.

Except for Accra, similar seasonal peaks in airborne pollen and fungal spores appear to be consistent across other major urban centres in Ghana. This suggests a strong overriding influence of regional climatic drivers associated with West African vegetation biomes and seasonal atmospheric circulation patterns. However, substantial local variation in fungal taxonomic composition is expected, reflecting differences in land use history, vegetation structure, and urbanization intensity. Together, these findings highlight both the importance of climatic forcing in shaping aerospora dynamics, and the current limitations imposed by the absence of long-term, standardized aerobiological monitoring networks in the region (Ajikah et al., 2021; Kalisa et al., 2019).

Côte d'Ivoire is similarly challenged by short-term surveys and limited research on aerial fungal spores. The majority of available data points suggest that the bioaerosol composition in Abidjan and its surrounding areas is representative of an amalgam of pollen from both local and introduced plant species, along with an abundant diversity of fungal spores, including *Cladosporium* and *Alternaria*. There remains a major gap with respect to creating comprehensive pollen calendars for Côte d'Ivoire; but the available data indicate that there are both summer and winter peaks and corresponding dry and wet formation and rebuilding periods of fungal spore production and Grass pollen release, respectively. Again, due to the lack of longitudinal studies, there are no comparable datasets or the ability to separate local climatic patterns from those events occurring worldwide. The methods used in previous aerobiological studies in West Africa–including both passive capture techniques and short-term volumetric trapping techniques– have significantly limited the ability to understand the regional dynamics of the aerospora, dating back to early coastal atmospheric mapping initiatives (Ybert, 1980). This has created significant challenges in linking umbilical factors, i.e. the weather/climate, to disease incidence/outcome. As a result of this inability to connect these important factors, estimates for the impact of climate change on the dynamics of the aerospora are solely speculative. This disadvantage will have particularly serious implications for the future as West Africa continues to undergo climate change, altering the distribution, season duration, and virulence of airborne fungal allergens and pathogens, which directly threatens public health systems (George et al., 2025).

### Eastern Africa

Eastern Africa covers a wide range of ecosystems—from equatorial rainforests and montane Afromontane forests at higher elevations, e.g. in Uganda, extensive savanna grasslands and dry woodlands influenced by seasonal rainfall, e.g. in parts of Tanzania, to semi‑arid dry forests and shrublands—shaped by climatic gradients, topography, and long‑term vegetation responses to climate variability to arid landscapes, e.g. in Somalia (Kalisa et al., 2019). Eastern Africa remains underrepresented in aerobiological research, with most studies conducted in Kenya, Uganda, and Ethiopia where scientific infrastructure is more developed. In Eastern Africa, only three studies have specifically addressed aerobiology, documenting airborne pollen and fungal spores in urban centres such as Nairobi, Kampala, and select locations in Ethiopia, highlighting the strong influence of seasonal rainfall and humidity on aeroallergen dynamics. Nairobi (Kenya) and Kampala (Uganda) have been the focus of early investigations, which indicate that grass pollen and fungal spores, particularly *Cladosporium* and *Alternaria*, dominate the atmospheric bioaerosol load. Peaks in both pollen and spore concentrations are strongly associated with the rainy seasons, reflecting the role of precipitation and humidity in spore release and pollen dispersal (Kiprop et al., 2024; Ismail et al., 1999).

In Ethiopia, available data are sparse and largely derived from short-term or localized surveys. Observations suggest that pollen from both indigenous plants and agricultural crops is prevalent, while fungal spores display strong temporal variability, often linked to seasonal rainfall and temperature fluctuations (Savina et al., 2025). The scarcity of long-term, standardized monitoring means that interannual trends and climate-driven changes in aerospora composition remain largely unknown.

**Fig. 6:**
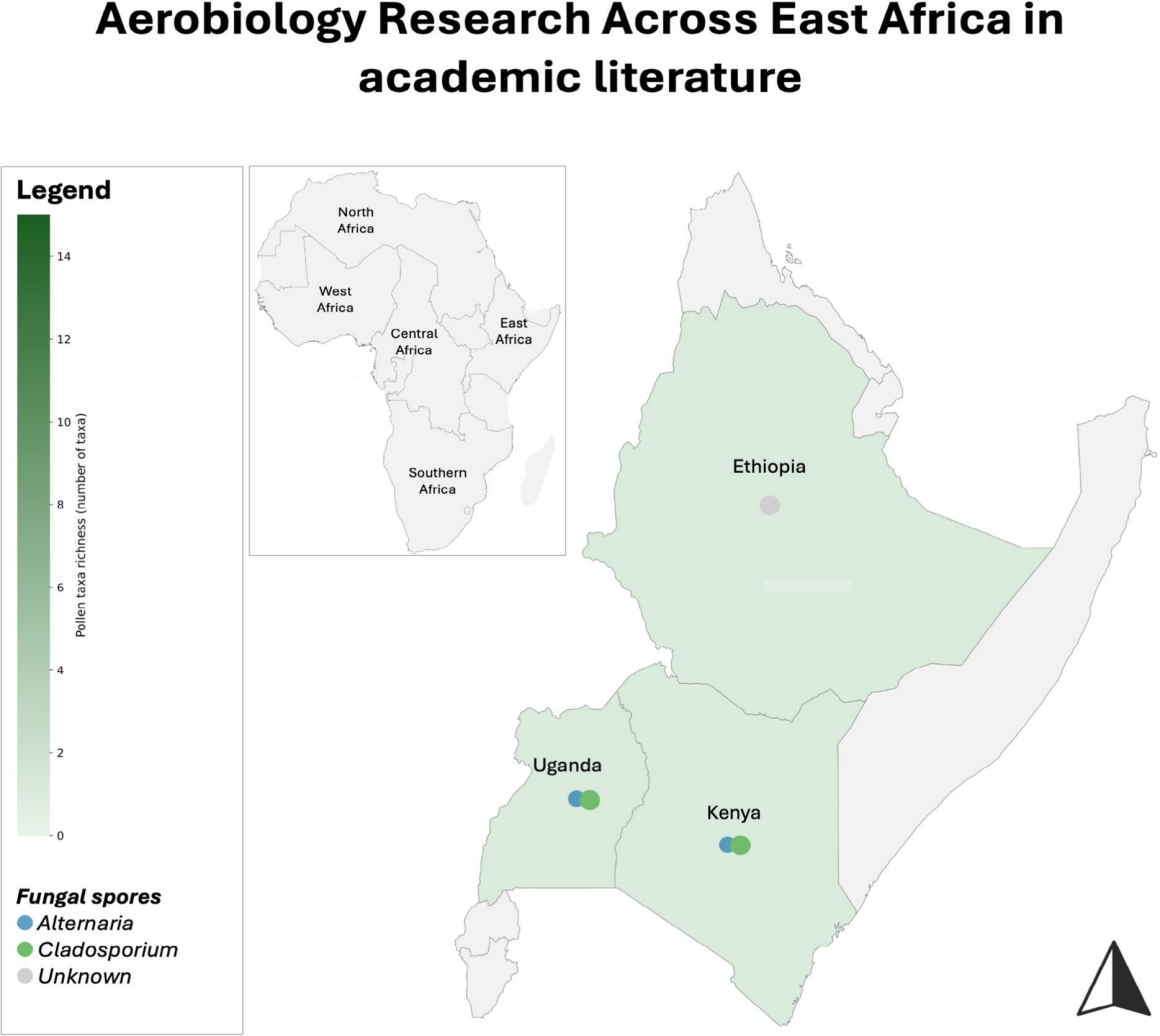
Map of aerobiology research in East Africa. The pollen taxa richness documented in the literature is indicated as a gradient of green along with the dominant fungal spores as coloured dots

Eastern Africa (Fig. 6) lacks continuous aerobiological monitoring, and existing studies are frequently limited in temporal and spatial scope. Establishing systematic sampling networks in major urban centres and integrating aerobiological, meteorological, and public health data are essential to understand the distribution and health impacts of aeroallergens in this region.

### Southern Africa

**Fig. 7:**
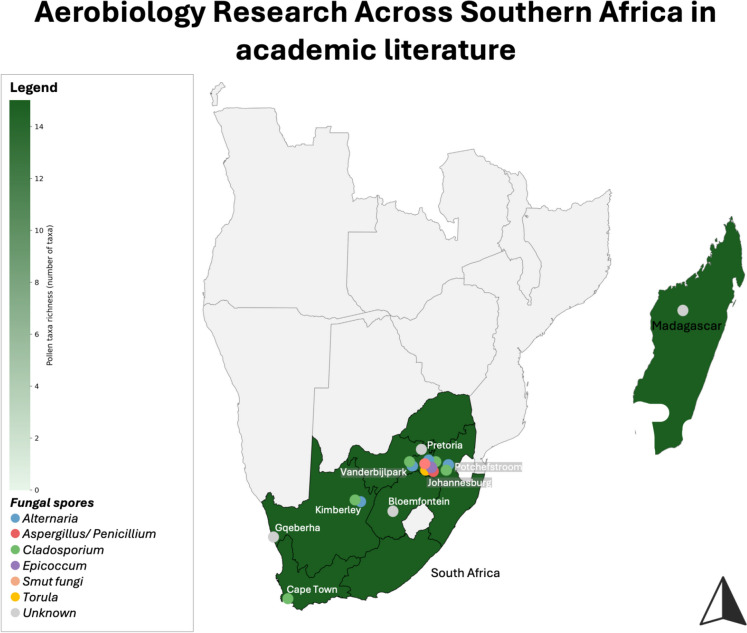
Map of aerobiology research in Southern Africa. The pollen taxa richness documented in the literature is indicated as a gradient of green, along with the dominant fungal spores indicated as coloured dots

Southern Africa supports a highly diverse mosaic of nine terrestrial biomes—including savannas (covering almost two thirds of the region in the North), grasslands (e.g. in northern South Africa and parts of Zimbabwe), forests, Mediterranean‑climate fynbos (restricted to the Cape region in South Africa) and Succulent Karoo shrublands (South Africa and neighbouring southern Namibia), semi‑arid Nama Karoo, and desert (mostly in Namibia, parts of Botswana and northwestern South Africa)—whose distribution and structure are shaped by gradients in rainfall seasonality, temperature regimes, fire, soil fertility, and other climatic drivers, resulting in one of the world’s most species‑rich and ecologically varied regions (Mucina & Rutherford, 2006, 2024). Southern Africa (Fig. 7) has the most developed aerobiological research infrastructure on the continent, anchored by South Africa's national monitoring network (SAPNET) and a decades-long monitoring record in Cape Town, alongside emerging research in Madagascar (Ajikah et al., 2020; Esterhuizen et al., 2023; Matuvhunye et al., 2026).

#### South Africa

Twenty-four references were identified in the South African aerobiology literature that specifically address airborne pollen and fungal spores, reflecting the historical and contemporary research underpinning our understanding of regional aeroallergens. South Africa has a rich history of aerobiological research, dating back to the mid-twentieth century, although systematic national monitoring is more recent, whereas studies from other southern African nations are rare (Berman, 2011, 2013, 2014; Ajikah et al., 2020). Early studies by Ordman and Etter in the 1950s and 1960s in Johannesburg and surrounding areas documented airborne pollen and fungal spores, establishing seasonal patterns and associations with meteorological factors (Ordman & Etter, 1956; Ordman, 1963). In the late twentieth century, researchers such as Cadman and Hawke conducted studies in Johannesburg, Pretoria, Durban, and Cape Town, measuring airborne pollen and fungal spores with volumetric samplers and exploring their relationship with meteorological variables (Cadman, 1990, 1991; Cadman et al., 1994, Cadman et al., 1997). These works identified Poaceae, Cupressaceae, and exotic ornamental trees as dominant pollen types, while fungal spores including *Alternaria* and *Cladosporium* were prevalent throughout the year. Although aerobiological monitoring in South Africa remained largely clinic-focused or city-specific until the 2010s, the establishment of the South African Pollen Network (SAPNET) in 2019 marked the first coordinated national effort, producing continuous airborne pollen and fungal spore data across multiple cities and biomes (Ajikah et al., 2020). These historical and modern datasets collectively provide baselines for understanding regional aerospora dynamics, seasonal trends, emerging aeroallergens such as *Ambrosia* (ragweed), and their clinical relevance in urban and peri-urban populations (Gharbi et al., 2024). Of the nine provinces of South Africa, only seven feature available aerobiological data that are summarized here. The high biodiversity is reflected in nine distinct biomes (Mucina & Rutherford, 2006). Although exotic trees are common in cities worldwide, South Africa hosts particularly high numbers due to favourable climatic conditions that support both temperate and subtropical species (Esterhuizen et al., 2023). Additionally, a recent study published by Matuvhunye et al., (2026) establishes the first multi-year, biome-specific baseline for airborne pollen exposure in South Africa. Led by the South African Pollen Monitoring Network (SAPNET), researchers tracked daily pollen concentrations across seven major cities over five years (2019–2024). The study reveals significant geographical differences: The highest overall pollen concentrations were recorded in the Grassland Biome (Bloemfontein), while the lowest were in the Albany Thicket Biome (Gqeberha).

***Gauteng province***

The smallest of the South African provinces is the economic hub of the country and harbours about 20% of the population. Consequently, the natural vegetation that is predominantly temperate Grassland Biome with patches of savanna on sheltered hilltops and rocky outcrops, is most affected by human impact, including intense urbanization with exotic plant invasion (Grobler et al., 2006; Mucina & Rutherford, 2006; Rutherford & Westfall, 1986). The province falls within the summer rainfall region with annual average monthly temperatures of 16,8 C and mean annual rainfall of 670 mm/year (Grobler et al., 2006). The vegetation comprises temperate grassland taxa such as *Themeda triandra* (red grass), *Eragrostis curvula* (weeping lovegrass) and *Aristida congesta* (three-awn grass), savanna elements including woody species like *Vachellia karroo* (sweet thorn) and *Dichrostachys cinerea* (sickle bush), and indicators of disturbance represented by exotic or ruderal species such as *Bidens pilosa* (blackjack) and *Tagetes minuta* (khaki weed), in cities diverse mostly exotic trees such as Jacaranda or plane trees are widespread (Esterhuizen et al., 2023; Grobler et al., 2006).

**Johannesburg**

Johannesburg has a warm-temperate climate with summer rainfall, characterized by warm, wet summers and dry, cool winters. It lies within the temperate Grassland biome, with patches of savanna, but these ecosystems are strongly influenced by urbanization (Mucina & Rutherford, 2006). Approximately 6.1% of the city is covered by trees, including remnant grassland and savanna vegetation, planted indigenous species, and naturalized alien species. Common taxa include *Platanus*, *Morus*, *Quercus*, *Jacaranda mimosifolia*, and *Eucalyptus* spp. A two-year daily sampling study in Johannesburg documented 85 aerospora taxa over August 2019–July 2021, with fungal spores dominating the assemblage (83.4% of counts) and including *Aspergillus/Penicillium*, *Cladosporium*, *Alternaria*, *Epicoccum*, *Torula*, and smut fungi among the most abundant (Roffe et al., 2025). Tree pollen (e.g. *Cupressus*, *Morus*, *Platanus*) and grass pollen (Poaceae) were additional major components. Rainfall, relative humidity, and temperature strongly influenced daily fungal spore and grass pollen counts, with specific combinations of moderate rainfall, mid‑range humidity, and temperatures of ~ 15–20 °C associated with higher aerospora levels during peak periods (Ajikah et al., 2023). Seasonality in Johannesburg mirrors broader regional trends: fungal spores tend to peak during moist and warmer periods, whereas pollen episodes often align with transitions to drier conditions. Moreover, early historical aerobiological records from the mid‑twentieth century and 1990s confirm that fungal spores have long dominated airborne bioaerosols, with grasses and neophyte tree pollen showing strong seasonal patterns (Cadman et al., 1997; Ajikah et al., 2020).

**Pretoria**

Pretoria, in the north of the province already within the Savanna Biome, is a SAPNET monitoring site since 2019; data show that grass pollen is a major component of the aerospora, with notable contributions also from tree (e.g. *Morus* (Mulberry tree)) and weed pollen types including exotic *Plantago*, and overall pollen spectra vary seasonally and differ even between nearby urban centres such as Johannesburg and Pretoria despite similar rainfall regimes, underscoring the influence of local vegetation and urban landscaping on aerobiology patterns (Esterhuizen et al., 2023). In Pretoria, *Morus*, *Betula*, *Ulmus*, *Platanus*, and Myrtaceae (probably mostly *Eucalyptus*) all reach > 3% of the API (Annual Pollen Index), underlining how dominant exotic trees mostly from the Northern Hemisphere are in South African cities as a source of pollen allergies (Esterhuizen et al., 2023). *Betula*, *Ulmus* and *Platanus* pollen are dispersed in September/October during the spring season, whereas *Morus* shows a second peak in November, and Myrtaceae pollen occur in comparatively lower numbers throughout the year (Esterhuizen et al., 2023). Grass pollen seasons tend to follow rainfall patterns with peak airborne concentrations typically from mid-summer into autumn (e.g. September to January/May) and tree pollen seasons peaking around early spring (starting late August and peaking in September), indicating a strong seasonality in aerospora concentrations that aligns with phenology and climate (Roffe et al., 2025).

**Vanderbijlpark**

In strongly industrialized and air-polluted Vanderbijlpark, to the South of Johannesburg in the Grassland Biome, similar focused studies are associated with the incidence of aerospora in the atopic population in a national air pollution hotspot. Gharbi et al. (2025a) showed that grass pollen was the most prevalent sensitizers, with *Cynodon dactylon* affecting 41.5% of participants, followed by *Lolium perenne* (ryegrass), a grass mix, and *Zea mays* (maize), each with a sensitization rate of 31.1%. Among tree pollen, *Olea* (olive) and *Platanus* were the most common, with 18.8% and 16.9% of participants sensitized, respectively. Weed pollen also contributed to allergic sensitization, particularly a mix of *Artemisia*, *Chenopodium*, *Salsola*, and *Plantago* (15.1%), and *Ambrosia* (10.3%). Fungal spores were less frequent, with *Alternaria* and *Cladosporium* affecting 8.5% and 4.7% of participants, respectively. Overall, the study highlights the dominant role of grass pollen, the significant contribution of allergens of exotic trees, and the presence of weed and fungal allergens in shaping the aeroallergen sensitization profile in this industrially impacted region.

***North West province***

Situated in the summer rainfall interior of South Africa, the province lies in the semi-arid to sub-humid Highveld and is, in the north, characterized by savanna, in the south by Grassland Biome. The climate has warm, wet summers and cool, dry winters, with mean annual rainfall of ~ 600–650 mm, supporting natural grassland to open savanna vegetation dominated by grasses such as *Themeda triandra*, *Eragrostis curvula* and *Heteropogon contortus*, remnants of which persist in urban nature reserves and along transport corridors, e.g. in the university city Potchefstroom where detailed botanical surveys were executed (Cilliers & Bredenkamp, 1998, 1999a, 2000). Urbanization and intensive agriculture and mining have caused strong disturbance, leading to fragmented and ruderal vegetation on road verges, railway reserves and vacant lots such as mine dumps, where exotic species such as *Morus alba*, *Tagetes minuta*, *Bidens pilosa* and *Pennisetum clandestinum* (Kikuyu grass) are common (Cilliers & Bredenkamp, 1998, 1999b, 1999c, 2000). Many exotic trees, such as *Platanus* and *Ulmus parviflora* (Chinese elm) grow as alley trees in Potchefstroom (Neumann et al., 2025).

**Potchefstroom**

Aerobiological monitoring in Potchefstroom, part of SAPNET, has been linked with clinical sensitization profiles. Air sampling was initiated in December 2022 using a 7‑day Hirst‑type volumetric spore trap mounted 12 m above ground at the North‑West University campus to continuously collect airborne biological particles (pollen grains and fungal spores). This monitoring followed international aerobiology standards and provided the first quantitative data on the local aerial pollen spectrum in this temperate grassland biome. Dominant airborne pollen types included grass pollen (Poaceae), tree pollen (e.g. *Platanus*, *Ulmus*, Cupressaceae, *Morus*, *Quercus*), and weed pollen, including *Ambrosia* (with the highest pollen counts within South Africa), with monthly peaks varying by taxon (e.g. grass in March, ragweed in March/April, plane and mulberry in September, elm in December) (Neumann et al., 2025). Overall, these data characterize the aerospora profile of Potchefstroom and highlight the presence of both indigenous and introduced allergenic pollen sources.

Complementing aerobiological sampling, a pilot health study conducted in 2024 evaluated allergic sensitization among 202 adults with respiratory symptoms in Potchefstroom using skin prick tests (SPTs) against an aeroallergen panel informed by local pollen monitoring. Main outcomes showed a high prevalence of sensitization to grass pollen (e.g. *Cynodon dactylon*, *Zea mays*) and tree pollen (*Platanus*, *Ulmus*), and fungal spores (*Alternaria*, *Cladosporium*). Over 90% of participants exhibited positive sensitization, with pollen as the most common trigger and notable correlations between allergic rhinitis symptoms and monthly pollen concentrations. These findings underscore the clinical importance of aerobiological monitoring in diagnosing and managing allergic diseases in South Africa (Gharbi et al., 2025b).

***KwaZulu-Natal province***

The province is temperate in the mountainous interior but (sub)tropical along the coast (Indian Ocean Coastal Biome), mostly affected by summer rainfall under the influence of the warm Agulhas Current with average summer temperatures fluctuating between c. 20 and > 35 C (Kloppers et al., 2020). Many tropical tree taxa reach their southernmost limit in the Indian Coastal Belt Biome (van Wyk, 1996) where rainfall is 1200-1500 mm/year and relative humidity is high (Mucina & Rutherford, 2006, 2024). The highly diverse vegetation encompasses mangroves and palmvelds but is also highly disturbed by human impact such as mining, agriculture (e.g. sugar cane plantations) and urbanization; exotic species in coastal KwaZulu-Natal include *Chromolaena odorata* (Triffid weed), *Acacia mearnsii* (Black wattle), and *Lantana camara* (Lantana) (Mucina & Rutherford, 2006).

**Durban**

At this site, pollen types contributing more than 3% to the API were dominated by Poaceae, followed by *Morus* spp., Polypodiaceae (common ferns), *Betula* spp., Asteraceae, Myrtaceae, *Cupressus*, and *Pinus* (Esterhuizen et al., 2023). Poaceae concentrations increased markedly in early summer, with smaller peaks from late summer to autumn, while arboreal pollen was present year-round. *Morus* peaked in late winter, *Betula* and Myrtaceae were elevated from spring to autumn, and *Cupressus* and *Pinus* showed higher abundances from late winter to late spring. Weed pollen increased mainly from summer to late autumn, with additional low-abundance taxa including *Ambrosia*, Cyperaceae, Urticaceae, *Artemisia*, and Chenopodiaceae (Esterhuizen et al., 2023; Gharbi et al., 2024).

***Eastern Cape province***

The Eastern Cape occupies a climatic transition zone where winter, all-year and summer rainfall generate high biodiversity, hosting eight out of the nine South African biomes including Fynbos, Savanna, Grassland, Nama Karoo and subtropical Albany Thicket with dense woody shrubs and succulents (Mucina & Rutherford, 2006; Tesfamichael et al., 2022). The Albany Centre of Plant Endemism in the coastal east is a biodiversity hotspot with rich, endemic vegetation adapted to temperate to subtropical conditions (Mucina & Rutherford, 2006). Rainfall and temperature gradients play a key role in shaping vegetation across the province, with remote-sensing studies revealing biome-specific vegetation responses to rainfall and land-surface temperature variability, highlighting complex climate–vegetation interactions in the Eastern Cape (Tesfamichael et al., 2022).

**Port Elizabeth**

Although long‑term local data are limited, Gqeberha has been included in the South African Pollen Monitoring Network (SAPNET) since 2019. The main pollen types recorded across the monitored cities were Poaceae, Cupressaceae, Moraceae, and *Buddleja*, reflecting both indigenous and introduced vegetation; patterns varied according to biome and season (Esterhuizen et al., 2023).

***Free State province***

**Bloemfontein**

Bloemfontein, located within the grassland biome but strongly affected by human impact including exotic trees and weeds, was also part of the first national aerospora monitoring effort by SAPNET (2019–2024). Bloemfontein exhibited high annual grass pollen concentrations, having the second-highest grass pollen integral in the country (trailing only the Savanna biome in Kimberley) with a five-year mean of 3,047 pollen* day/m^3^ (Matuvhunye et al., 2026).

***Northern Cape province***

**Kimberley**

In Kimberley, the aerobiological spectrum is characterized by dominant Poaceae pollen with peak airborne concentrations typically occurring during the summer rainfall season, and smaller contributions from tree pollen such as Oleaceae (olive family); weed pollen levels are generally low compared with grasses and trees at this site. Fungal spores—including common aeroallergens like *Cladosporium* and *Alternaria*—are also present and fluctuate with humidity and weather conditions, although detailed long‑term fungal spore seasonality is still being refined (Esterhuizen et al., 2023). Continuous monitoring tracks emerging aeroallergens, such *as Ambrosia* pollen, which has been detected sporadically in Kimberley and is monitored due to its high allergenic potential and possible range expansion (Gharbi et al., 2024).

***Western Cape province***

The Western Cape has a Mediterranean climate, with hot, dry summers and cool, wet winters; inland areas may drop below freezing at night. Summer temperatures range from 15–27 °C, winter from 5–22 °C, and rainfall occurs mainly in winter (300–1000 mm along the coast, less inland) (Botai et al., 2017). Vegetation is dominated by the highly diverse Fynbos Biome (dominant families Ericaceae, Proteaceae, Restionaceae), with Succulent Karoo in drier interior regions and the north (Mucina & Rutherford, 2006). Small patches of Afrotemperate forest (e.g. Podocarpus) occur along the southern coast and mountains. Dominated airborne pollen originates from many alien trees—including *Quercus, Pinus, Populus, Platanus, Eucalyptus*, and *Acacia*—which were introduced historically by Dutch settlers since the seventeenth century, alongside crops like wheat, apples, peaches, and oranges (Esterhuizen et al., 2023).

**Cape Town**

Aerobiology research in southern Africa was initiated by the Aerobiologist David Ordman from 1945 to 1972. He noted that dominant Cupressaceae (Ordman, 1945) and Poaceae (Ordman, 1964) were found to be a source of sensitization in various regions of the country. In addition, earlier studies in Cape Town from the late twentieth century documented the persistent dominance of fungal spores such as *Cladosporium* and Poaceae pollen in the atmosphere, with relationships to local meteorological conditions, including wind, humidity, and rainfall (Hawke & Meadows, 1989). Afterwards, Potter and colleagues further contributed to long-term monitoring in the Western Cape, supporting clinical allergy research (Potter et al., 1991). As a result, Berman and Potter’s decades-long work in Cape Town established one of the longest continuous aerobiological datasets in the Southern Hemisphere (Berman, 2018). Berman compiled approximately 30 years of volumetric trap data, linking airborne pollen and fungal spores with clinical assessments of allergic respiratory disease. These historical records provide valuable baselines for interpreting contemporary SAPNET data and highlight long‑standing aeroallergen dynamics in coastal Mediterranean‑type climates (Berman, 2018). Additionally, the Western Cape provides an example of aerobiology integrated with respiratory health research. A panel study among schoolchildren in informal settlements measured ambient concentrations of *Alternaria* and *Cladosporium* spores using spore traps in parallel with repeated lung function testing, demonstrating that daily fluctuations in ambient fungal spores can independently affect lung function in children and underscore the clinical relevance of aerobiological monitoring in the region (Olaniyan et al., 2020).

#### Madagascar

Madagascar features a tropical–subtropical climate with strong east–west and north–south rainfall gradients driven by topography, easterly trade winds, the northwesterly monsoon, and the seasonal passage of the ITCZ (Jury, 2016; Tadross et al., 2008). The combination of climatic and topographic conditions, along with the island’s isolation, has resulted in a diverse environment ranging from tropical rainforest to grasslands and spiny thickets (Antonelli et al., 2022). The island is considered a biodiversity hotspot with high species richness and exceptional levels of endemism across many taxonomic groups, combined with high rates of habitat degradation and fragmentation (Meyers et al., 2000). Twenty-two studies have specifically addressed aerobiology, including airborne pollen monitoring, aeropalynology, and clinical aeroallergen investigations, providing key insights into the distribution, seasonality, and allergenic potential of pollen across the island, while additional palynology studies—including research on pollen morphology, quaternary vegetation, and melissopalynology—are also cited in this section to provide broader context on the island’s plant and pollen diversity.

The first palynological studies (1966–1988) looking at pollen morphology, characterization and quaternary were conducted by Straka (Straka & Simon, 1967; Straka et al., 1967; Straka & Friedrich, 1982, 1983, 1984, 1988), and since then the field has expanded across multiple disciplines including aeropalynology, melissopalynology, and palaeoecology with major developments occurring in the last decade (e.g. Vololona et al., 2019; Razanatsoa et al., 2022; Razafimanantsoa et al., 2024, 2025). A recent study by Razafimanantsoa and Razanatsoa (2024) demonstrated that pollen rain reflects the main vegetation types across Madagascar and provided information on the current structure and state of the landscape, including open ecosystems, degraded and anthropogenic impacts such as plantations. Aeropalynological infrastructure for pollen monitoring in Madagascar is limited, with two traps located in the Central Highlands (installed in the 1980s) and in the northwestern region of Madagascar (installed in 2023); annual records exist, but no continuous records spanning multiple years are available. However, research produced from these facilities has largely been based on postgraduate theses (e.g. Andriamanampisoa, 2025; Andriantsitoha, 2008; Rabarisoa, 2007; Rakotonirina, 2008; Rakotoson, 2007; Rasolofonirina, 2025; Razafimelison, 2008), which, in combination with clinical studies on airborne pollen and spores (e.g. Ramavovololona et al., 2013; Randrianandraina et al., 2022), have nonetheless made important contributions to understanding aerospora on the island.

***Central highlands***

The Central Highlands consist of a wooded grassland–bushland mosaic with fragmented sub-humid forests between 800 and 2000 m asl, mainly in ravines and along rivers (Moat & Smith, 2007). Grasslands dominate (> 70%) and are interspersed with limited native woody taxa and scattered introduced species such as *Eucalyptus*, *Pinus*, and *Acacia* (Humbert, 1955; Vorontsova et al., 2016). The presence of large marshes with Cyperaceae and Nymphaeaceae is also noteworthy. A first aeropalynological study was conducted in Antananarivo, the capital of Madagascar, in 1984 (Rajeriarison, 1984). The research examined how different vegetation types and climatic factors influenced atmospheric pollen composition. Using a COUR pollen trap, pollen was collected over three years (1979–1981) to analyze pollen flow patterns (Rajeriarison, 1984). This work was later expanded through the installation of Hirst pollen traps at three experimental stations in Antananarivo, Ambatondrazaka, and Antsirabe (Andriantsitoha, 2008; Rabarisoa, 2007; Rakotonirina, 2008; Rakotoson, 2007; Razafimelison, 2008) with the goal of collecting data on atmospheric pollen composition across the Malagasy Highlands. During this investigation, species such as *Cupressus* sp. and *Casuarina equisetifolia* characterizing plantations, *Rhynchelytrum repens, Cynodon dactylon r*epresenting grasslands and savanna*, Zea mays* abundant in agricultural landscapes, and Amaranthaceae family often associated with disturbed areas were the most abundant taxa. Clinical and immunological investigations found that grass species *Imperata cylindrica, Rhynchelytrum repens, Panicum maximum, Pennisetum polystachion, Cynodon dactylon* and *Aristida rufescens* have a high impact on allergic occurrence (Ramavovololona et al., 2013). A study covering three years (2005, 2006, 2008) in Antananarivo with comparison of pollen influx with meteorological data found that herbaceous pollen dispersal, mostly Poaceae, take place during the hot and rainy season, while pollen emissions of trees and shrubs are especially during the dry and fresh season (Andriamahery, 2014). Over 80 pollen types were identified, with thirteen taxa occurring consistently. These taxa reflect Central Highlands vegetation, including *Pinus* spp.**,** *Cynodon dactylon***,** *Rhynchelytrum repens***,** *Eucalyptus* sp.**,** *Panicum maximum***,** *Cupressus* sp.**,** *Casuarina equisetifolia***,** *Macaranga* sp.**,** and *Cyperus* spp**.** The study recorded woodland-associated species such as *Podocarpus* sp.**,** *Trema orientalis* and *Fraxinus* sp. (Andriamahery, 2014).

***Northwest Region***

The Northwest region, the second region to benefit from aerobiological research, is characterized by dry deciduous forest combined with diverse vegetation such as savannas, the most common of which are shrub savannas with *Ziziphus* sp., *Medemia* sp., and *Acridocarpus* sp., mangroves, and swamp vegetation (Moat & Smith, 2007), all of which constitute potential sources. The initial study focuses on the atmospheric pollen emissions from various vegetation using a Cour Sampler trap (Ramavovololona, 1986). Multiple allergenic species such as *Rhynchelytrum repens, Aristida rufescens, Cynodon dactylon, Pennisetum polystachium, Imperata cylindrica, Zea mays, Oryza sativa, Panicum maximum,* and *Helianthus annuus,* mostly found in savannas resp. agricultural areas, were recorded (Ramavovololona, 1998). These species were also recorded in the main city of Mahajanga (Randrianjafy, 2018), with some additional species such as *Aristida rufescens, Allocasuarina littoralis, Tithonia diversifolia, Mangium,* and *Eucalyptus maculata* suggested by local communities to be allergenic (Rakotomamonjy, 2016).

Recently, in April 2023, the city of Mahajanga acquired a Burkard-type Hirst volumetric pollen trap allowing the establishment of a continuous pollen monitoring program. Despite its recent installation, data are emerging with a postgraduate thesis being finalized (e.g. Andriamanampisoa, 2025; Rasolofonirina, 2025). Preliminary results from April 2023 to April 2024 identified 18 distinct pollen types, of which 89% were classified as allergenic taxa. These include *Amaranthus viridis*, *Tridax procumbens*, *Acmella oleracea*, *Acalypha indica*, *Cynodon dactylon*, *Agropyron repens*, *Zea mays*, and cf. *Panicum maximum*. The remaining 11% were categorized as non-allergenic and include *Casuarina equisetifolia*, *Eucalyptus maculata*, *Wedelia trilobata*, *Araucaria heterophylla*, *Peltophorum pterocarpum*, *Aira praecox*, and *Ixora coccinea*. Ethnobotanical and vegetation surveys conducted around four Fokontany (the smallest administrative unit in Madagascar) in Mahajanga found that, among twenty monitored plant species, sixteen were reported as allergenic by participants. Of these, six species were also detected in the Burkard-type pollen trap. These six species are *Albizia lebbeck*, *Anacardium occidentale*, *Cynodon dactylon*, *Eucalyptus maculata*, *Zea mays*, and *Casuarina equisetifolia* (Rasolofonirina, 2025). Moreover, no significant correlations have been found between the meteorological parameters and the pollen flux for this period (Andriamanampisoa, 2025). Investigation regarding the exposure of patients to sugarcane (*Saccharum officinarum*) pollen is associated with clinical manifestations of allergic rhinitis and confirms the role of this allergen in the occurrence of the disease (Randrianandraina et al., 2022). In collaboration with the local medical centre, Centre Médical Falivanja, which specializes in allergies and respiratory diseases, ongoing research is investigating the relationship between pollen recorded in the traps and allergy tests performed at the centre. This includes tests using extracts of *Cynodon dactylon* and *Aristida*, the most common Poaceae species in the city of Mahajanga and the Boeny region and the development of new tests for tree pollen, for which no tests currently exist.

### Comparative synthesis across African regions

A continent-wide comparison of aerobiological data reveals marked spatial and temporal variation in pollen and fungal spore dynamics, largely shaped by climate, vegetation type, and anthropogenic factors. North Africa, with its Mediterranean climate, exhibits well-defined pollen seasons for olive, cypress, and grasses, alongside seasonal fungal peaks of *Cladosporium* and *Alternaria*. Central Africa, dominated by tropical rainforests, displays high taxonomic diversity in both pollen and fungal spores, but systematic, long-term monitoring is virtually absent. West Africa shows pronounced seasonal contrasts, with fungal spores peaking in the rainy season and pollen concentrations rising during dry Harmattan periods, especially in Nigeria. Eastern Africa’s limited datasets highlight grass pollen and fungal spore dominance during rainy seasons, but temporal and spatial coverage remains insufficient. Southern Africa benefits from advanced monitoring networks, notably SAPNET, which provide detailed seasonal and meteorological correlations, and link aeroallergen exposure to public health outcomes.

Across all regions, climatic factors—including rainfall, temperature, humidity, and wind patterns—emerge as primary drivers of aerospora variability. Vegetation structure and land use further modulate pollen and spore composition, while urbanization and agricultural practices influence local concentrations. Major research gaps persist, including the lack of standardized, long-term monitoring in Central, Eastern, and much of West Africa, limited aeromycological data in Egypt and tropical regions, and sparse health-linked studies outside South Africa and Nigeria. While countries such as South Africa and Nigeria have developed substantial datasets linking aerospora dynamics to climate variability and public health outcomes, large parts of Central, Eastern, and West Africa remain effectively unmonitored.

The limited number and uneven geographical distribution of aerobiological studies across Africa reflects a combination of societal, economic, infrastructural, and geographical/climatic factors. In many African countries, lower levels of gross domestic product (GDP) and constrained funding for scientific research may limit investment in long-term environmental monitoring programmes (Patra & Muchie, 2021), including aerobiological surveillance. In addition, under-resourced health systems and competing public health priorities—particularly the burden of infectious diseases—may reduce attention and funding directed towards allergic diseases and airborne biological monitoring. Differences in educational and research capacity, including limited availability of specialized training, technical expertise, laboratory facilities, and monitoring equipment, may further contribute to reduced research output in aerobiology. Furthermore, climatic and geographical heterogeneity across the continent may also influence research priorities and study feasibility. Regions characterized by arid climates, extreme seasonal variability, or limited vegetation cover may experience different aerobiological dynamics, while logistical challenges associated with remote or under-monitored areas can further restrict systematic data collection. Collectively, these factors may partly explain the observed gaps in aerobiological knowledge across many African regions. Filling these gaps through continent-wide, harmonized aerobiological surveillance, integrated with meteorological and clinical data, is essential to improve understanding of aeroallergen dynamics, inform public health policies, and anticipate the effects of climate change on airborne pollen and fungal spores in Africa.

## Conclusion

This review demonstrates that aerobiological research in Africa remains highly uneven in both geographic coverage and methodological standardization. While countries such as South Africa and Nigeria have developed substantial datasets linking aerospora dynamics to climate and public health outcomes, large parts of Central, Eastern, and West Africa remain effectively unmonitored.

Across the continent, airborne pollen and fungal spore dynamics are primarily driven by climatic factors—rainfall, temperature, humidity, and wind regimes—interacting with vegetation structure, land-use change, and urbanization. Grass pollen and fungal spores (notably *Cladosporium* and *Alternaria*) emerge as dominant aeroallergens in most African regions, with increasing evidence of sensitization and respiratory health impacts. Addressing existing knowledge gaps will require the establishment of coordinated long-term aerobiological monitoring networks, harmonized methodologies, and closer integration with meteorological data and clinical allergy surveillance. Such efforts are essential for developing pollen calendars, forecasting allergen exposure, and mitigating the growing burden of allergic respiratory disease under ongoing climate change and rapid socio-environmental transformation across Africa.

## Data Availability

No datasets were generated or analyzed during the current study.

## References

[CR1] Aboulaich, N., Achmakh, L., Bouziane, H., Trigo, M. M., Recio, M., Kadiri, M., Cabezudo, B., Riadi, H., & Kazzaz, M. (2013a). Effect of meteorological parameters on *Poaceae* pollen in the atmosphere of Tétouan (NW Morocco). *International Journal of Biometeorology,**57*(2), 197–205. 10.1007/s00484-012-0566-2 22744802 10.1007/s00484-012-0566-2

[CR2] Aboulaich, N., Bouziane, H., Kadiri, M., & Riadi, H. (2008). Male phenology and pollen production of Cupressus sempervirens in Tetouan (Morocco). *Grana,**47*(2), 130–138. 10.1080/00173130802151700

[CR3] Aboulaich, N., Bouziane, H., Kadiri, M., Trigo, M. M., Riadi, H., Kazzaz, M., & Merzouki, A. (2009). Pollen production in anemophilous species of the Poaceae family in Tetouan (NW Morocco). *Aerobiologia,**25*, 27–38. 10.1007/s10453-008-9106-2

[CR4] Aboulaich, N., Trigo, M. M., Bouziane, H., Cabezudo, B., Recio, M., El Kadiri, M., & Ater, M. (2013b). Variations and origin of the atmospheric pollen of *Cannabis* detected in the province of Tetouan (NW Morocco): 2008–2010. *Science of the Total Environment,**443*, 413–419. 10.1016/j.scitotenv.2012.10.075 23208276 10.1016/j.scitotenv.2012.10.075

[CR5] Achmakh, L., Bouziane, H., Aboulaich, N., Trigo, M. M., Janati, A., & Kadiri, M. (2015). Airborne pollen of *Olea europaea* L. in Tetouan (NW Morocco): Heat requirements and forecasts. *Aerobiologia,**31*(2), 191–199. 10.1007/s10453-014-9356-0

[CR6] Achmakh, L., Janati, A., Boullayali, A., ElHassani, L., & Bouziane, H. (2020). Forecasting olive (Olea europaea L.) production using aerobiological and meteorological variables in Tétouan (NW Morocco). *Aerobiologia,**36*(4), 749–759. 10.1007/s10453-020-09665-5

[CR7] Adekanmbi, O. H., Alebiosu, O. S., & Adeiga, A. A. (2019). Aerofloral investigation and allergenic potentials of two dominant airborne pollen types at selected sites in south-western Nigeria. *Aerobiologia,**35*(1), 27–44. 10.1007/s10453-018-9533-7

[CR8] Adeniyi, T. A., Adeonipekun, P. A., & Olowokudejo, J. D. (2018a). Annual records of airborne pollen of Poaceae in five areas in Lagos. *Nigeria. Grana,**57*(4), 284–291. 10.1080/00173134.2017.1356865

[CR10] Adeniyi, T. A., Adeonipekun, P. A., Olowokudejo, J. D., & Akande, I. (2018b). Allergenicity of dominant aeropollen in Nigeria (Part II). *Current Allergy & Clinical Immunology,**31*(3), 178–183.

[CR9] Adeniyi, T. A., Adeonipekun, P. A., Olowokudejo, J. D., & Akande, I. S. (2014). Airborne pollen records of Shomolu local government area in Lagos State. *Notulae Scientia Biologicae.,**6*(4), 428–432.

[CR187] Adeniyi, T. A., Adeonipekun, P. A., Olowokudejo, J. D., & Akande, S. I. (2017). Allergenicity of dominant aeropollen in Nigeria. Part 1. *Current Allergy and Clinical Immunology, 30*(4), 264–269.

[CR11] Adeonipekun, A. P. (2012). Comparative aeropalynology of Ota, Nigeria. *Journal of Ecology and the Natural Environment,**4*(12), 314–320.

[CR12] Adeonipekun, P. A., & John, B. A. (2011). Airborne pollen and fungal spores of South-Western Nigeria: Implications for allergy sufferers. *Grana,**50*(2), 130–140.

[CR13] Adeonipekun, P. A., John, B. A., & Oyebamiji, N. A. (2020). Aerobiology of pollen and fungal spores in tropical environments: Implications for allergy research in Nigeria. *Aerobiologia,**36*, 603–617.

[CR185] Adomou, A. C. (2005). Vegetation Patterns and Environmental Gradients in Benin: Implications for Biogeography and Conservation. Ph.D. Thesis, Wageningen University, Wageningen, The Netherlands. 133 pp. ISBN: 90-8504-308-5. 10.18174/121707

[CR14] Afif, H., Mokahli, S., Bourra, H., Aichane, A., & Bouayad, Z. (2006). Sensibilisation cutanée au cyprès à Casablanca Cutaneous sensitisation to cypress in Casablanca. *Revue Française D’allergologie Et D’immunologie Clinique,**46*, 633–639.

[CR15] Agwu, C. O. C., & Osibe, E. E. (1992). Airborne palynomorphs of Nsukka during the months of February–April, 1990. *Nigerian J Bot,**5*, 177–185.

[CR16] Ajikah, L. B., Alebiosu, O. S., Adekanmbi, O. H., & Oshinlaja, E. O. (2017). Aeropalynological investigation of three local governments in Lagos. *Southwest Nigeria. Nigerian Journal of Botany,**30*(7), 107–118.

[CR195] Ajikah, L., Neumann, F.H., Berman, D., & Peter, J. (2020). Aerobiology in South Africa: A new hope! *South African Journal of Science, 116*(7–8), 1–4. 10.17159/sajs.2020/8112

[CR17] Ajikah, L., Ogundipe, O. T., & Bamgboye, O. (2015). Palynological survey of airborne pollen and spores in the University of Lagos, Akoka campus, southwestern Nigeria. *IFE Journal of Science,**17*(3), 643–655.

[CR18] Ajikah, L. B., Roffe, S. J., Neumann, F. H., Bamford, M. K., Esterhuizen, N., Berman, D., & Peter, J. (2023). Meteorological influences on airborne pollen and spores in Johannesburg (Gauteng), South Africa. *Aerobiologia,**39*, 363–388. 10.1007/s10453-023-09799-2

[CR19] Ajikah, L. B., Shadrak Alebiosu, O., Orijemie, E. A., & Onah, D. (2021). A review of aeropalynology research in Nigeria: Implications on public health and environmental research collaboration. *Allergologia Et Immunopathologia,**49*(6), 31–38. 10.15586/aei.v49i6.241 34761653

[CR20] Ajouray, N., Bouziane, H., Trigo Pérez, M. M., & Kadiri, M. (2016). Variation interannuelle des spores fongiques de Tétouan (Nord-Ouest du Maroc) et calendrier sporal. *Journal De Mycologie Médicale,**26*(2), 148–159. 10.1016/j.mycmed.2016.02.018 26994760 10.1016/j.mycmed.2016.02.018

[CR21] Akasoro, O. M., Sowunmi, A. M., & Adeonipekun, P. A. (2026). Airborne pollen and spores of the University of Ibadan Campus, Ibadan. *Southwest Nigeria. Aerobiology,**2026*(4), 10.

[CR183] Akoègninou, A. (1984). Contribution à l’étude botanique des ilôts de forêt dense humide semo-décidue en République Populaire du Bénin. Thèse 3éme cycle. Univ. Bordeaux E7. 250p

[CR184] Akoègninou, A., Van der Burg, W., & Van der Maesen, L. J. G. (2006). Flore analytique du Bénin. Backhuys Publishers, Wageningen, 1063 pp.

[CR22] Amroune, A., Mohamdi, N., Ben Moussa, M. T., Gharbi, D., & Smati, D. (2026). First pollen calendar for Batna and its surrounding areas: results of an aeropalynological study conducted during the pollen year year 1 November 2022–31 October 2023. *Batna Journal of Medical Sciences.,**13*(2), 8.

[CR23] Andriamahery, Y. H., 2014. Émissions polliniques atmosphériques d’Antananarivo année 2008. Comparaison avec les données de 2005 et 2006. Mémoire (DEA) en Biologie et Écologie Végétales option Palynologie Appliquée, Univ. Antananarivo, 50 p.

[CR24] Andriamanampisoa, J. F. R. (2025). *Contenu et dynamisme des pollens atmosphériques dans la ville de Mahajanga* (p. 67). Université de Mahajanga.

[CR25] Andriantsitoha, D., 2008. Émission pollinique, phénologie de la floraison et facteurs climatiques dans la région d’Antsirabe pour les années 2004–2005 et 2005–2006. Mémoire de D.E.A. Palynologie Appliquée, Faculté des Sciences, Université d’Antananarivo, 140 p.

[CR26] Antonelli, A., Smith R. J., Perrigo A.L., Crottini A., Hackel J., Testo W., Farooq H., et & al. 2022. Madagascar’s Extraordinary Biodiversity: Evolution, Distribution, and Use. *Science* 378(6623): eabf0869. 10.1126/science.abf0869. 10.1126/science.abf0869 36454829

[CR27] Bardei, F., Bouziane, H., Kadiri, M., Rkiek, B., Tebay, A., & Saoud, A. (2016). Profils de sensibilisation cutanée aux allergènes respiratoires des patients de la ville de Tétouan (Nord Ouest du Maroc). *Revue De Pneumologie Clinique,**72*(4), 221–227. 10.1016/j.pneumo.2016.04.005 27349826 10.1016/j.pneumo.2016.04.005

[CR28] Bardei, F., Bouziane, H., Trigo Perez, M. M., Ajouray, N., El Haskouri, F., Filali Ben Sidel, F., Abiri, R., Kadiri, M., Kazzaz, M., & Riadi, H. (2013). Incidence des spores fongiques de l’air de Tétouan (NW du Maroc) et influence des paramètres météorologiques. *Revue Française D’allergologie,**53*(7), 576–584. 10.1016/j.reval.2013.05.004

[CR29] Bassiouni, E. M., & Taia, W. K. (2021). Study of the airborne pollen and spores in the atmosphere of Alexandria City. *Egypt. Acta Scientific Microbiology,**4*, 38–49.

[CR134] Batna Pollen. (n.d.). *BATNA POLLEN: Batna, Allergies Polliniques Soignées*. https://batna-pollen.combatna-pollen

[CR30] Ben Dhiab, A., Ben Mimoun, M., Oteros, J., García-Mozo, H., Domínguez-Vilches, E., Galán, C., Abichou, M., & Msallem, M. (2017). Modeling olive-crop forecasting in Tunisia. *Theoretical and Applied Climatology,**128*(3), 541–549.

[CR32] Berman, D. (2011). Climate change and aeroallergens in South Africa. *Current Allergy & Clinical Immunology,**24*(2), 65–71.

[CR33] Berman, D. (2013). Regional-specific pollen and fungal spore allergens in South Africa. *Current Allergy & Clinical Immunology,**26*(4), 196–209.

[CR34] Berman, D. (2014). Aerobiology studies in South Africa. *Current Allergy & Clinical Immunology,**27*(4), 255–258.

[CR31] Berman, D. (2018). Variations in pollen and fungal air spora: An analysis of 30 years of monitoring for the clinical assessment of patients in the Western Cape (PhD thesis). Cape Town: University of Cape Town.

[CR35] Botai CM, Botai JO, de Wit JP, Ncongwane KP, Adeola AM (2017) Drought Characteristics over the Western Cape Province, South Africa. *Water*, *9*(11): 876. 10.3390/w9110876

[CR36] Bouaissa, O. (2026). Sensibilisation aux allergènes polliniques au Maroc: Revue des données cliniques publiées de 2000 à 2025. *Revue Française D’allergologie,**66*(1), Article 104639. 10.1016/j.reval.2025.104639

[CR37] Boullayali, A., Ater, M., Terral, J. F., & Bouziane, H. (2024). Comparison of *Olea* pollen sampling between gravimetric and volumetric traps (NW of Morocco). *Science of the Totalenvironment,**951*, Article 175663. 10.1016/j.scitotenv.2024.175663 10.1016/j.scitotenv.2024.175663 39173772

[CR38] Boullayali, A., Elhassani, L., Janati, A., Achmakh, L., & Bouziane, H. (2021). Airborne pollen trends in Tétouan (NW of Morocco). *Aerobiologia,**37*(3), 479–505.

[CR39] Boullayali, A., Galán, C., Martínez-Bracero, M., & Bouziane, H. (2023). Chilling and heat requirements for woody taxa in Tétouan (NW Morocco). *Aerobiologia,**39*(2), 241–255. 10.1007/s10453-023-09789-4

[CR40] Boullayali, A., Hassoun, M., & Bouziane, H. (2025). Variation of pollen season trends under Mediterranean climate: A systematic review. *Aerobiologia,**41*, 469–488. 10.1007/s10453-025-09862-0

[CR41] Boullayali, A., Raissouni, I., Sahli, A., & Bouziane, H. (2026). Methodological comparison of pollen season definitions and temporal trends in allergenic taxa (2008–2024). *Aerobiologia,**42*, 26. 10.1007/s10453-026-09915-y

[CR42] Bounil, S., Mezenner, H., Naamoune, S., & Salah, S. S. (2022). Prévalence et phénotype clinique des allergies respiratoires dans une région du centre algérien. *Revue Française D’allergologie,**62*(3), 294. 10.1016/j.reval.2022.02.014

[CR43] Bouziane, H., Raissouni, I., Bouziane, I., El Bakali, S., & Sakar, H. (2025). Estimation of chilling and heat accumulation periods of the olive tree in a warm and sub-humid climate, using the partial least squares (PLS) regression. *Aerobiologia,**41*(3), 667–679. 10.1007/s10453-025-09873-x

[CR44] Buters, J. T. M., Antunes, C., Galveias, A., Bergmann, K. C., Thibaudon, M., Galán, C., Schmidt-Weber, C., & Oteros, J. (2018). Pollen and spore monitoring in the world. *Clinical and Translational Allergy,**8*(1), 9. 10.1186/s13601-018-0197-8 29636895 10.1186/s13601-018-0197-8 PMC5883412

[CR46] Cadman, A. (1990). Airspora of Johannesburg and Pretoria, South Africa, 1987/88: I. *Pollen Calendars. Grana,**29*(4), 311–317. 10.1080/00173139009428942

[CR47] Cadman, A. (1991). Incidence of atmospheric pollen in the Pretoria Witwatersrand-Vereeniging region during 1987/1988. *South African Medical Journal,**79*(1), 84–87. 1989094

[CR48] Cadman, A., & Dames, J. F. (1994). Airspora of Durban: A sub-tropical coastal South African city. *Grana,**90*, 607–610.

[CR49] Cadman, A., Dames, J. F., Terblanche, P. S., & Nel, R. (1997). The AIRKEM study in Gauteng, South Africa: The role of the airspora in an industrial urban environment. *Grana,**36*(3), 175–179. 10.1080/00173139709362605

[CR50] Cambon, G. (1981). *Relation entre le contenu pollinique de l’atmosphère et le couvert végétal en Méditerranée occidentale à Montpellier (France), Valence (Espagne), et Oran (Algérie)* (p. 105). Université des Sciences et Techniques du Languedoc, Montpellier.

[CR51] Catarino, L., & Romeiras, M. M. (2020). Biodiversity of vegetation and flora in tropical Africa. *Diversity,**12*(10), 369.

[CR52] Chahat, N., Bouguenoun, I., Bouguenoun, W., & Houhamdi, M. (2017). Contribution to the quantitative and qualitative study of atmospheric pollens (Guelma, Northeast of Algeria). *World Journal of Environmental Biosciences,**6*(3), 8–16.

[CR53] Cilliers, S., & Bredenkamp, G. J. (1998). Vegetation of railway reserves in the Potchefstroom municipal area, North West Province, South Africa. *South African Journal of Botany,**64*, 271–280.

[CR54] Cilliers, S. S., & Bredenkamp, G. J. (1999a). Urban nature conservation: Vegetation of natural areas in the Potchefstroom municipal area, North West Province, South Africa. *Koedoe,**42*, 1–30.

[CR55] Cilliers, S. S., & Bredenkamp, G. J. (1999b). Analysis of the spontaneous vegetation of intensely managed urban open spaces in the Potchefstroom Municipal Area, North West Province, South Africa. *South African Journal of Botany,**65*, 59–68.

[CR56] Cilliers, S. S., & Bredenkamp, G. J. (1999c). Ruderal and degraded natural vegetation on vacant lots in the Potchefstroom Municipal Area, North West Province, South Africa. *South African Journal of Botany,**65*, 163–173.

[CR57] Cilliers, S. S., & Bredenkamp, G. J. (2000). Vegetation on road verges on an urbanisation gradient in Potchefstroom, South Africa. *Landscape and Urban Planning,**46*, 217–239.

[CR58] Clot, B., Gilge, S., Hajkova, L., et al. (2024). The EUMETNET AutoPollen programme: Establishing a prototype automatic pollen monitoring network in Europe. *Aerobiologia,**40*(1), 3–11.

[CR59] D’Amato, G., Pawankar, R., Vitale, C., Ansotegui, I., Rosario, N., Haahtela, T., Galán, C., Murrieta-Aguttes, M., Cecchi, L., Bergmann, C., Ridolo, E., Bergmann, K. C., D’Amato, M., & Annesi-Maesano, I. (2020). Climate change and allergic diseases in tropical and subtropical regions. *World Allergy Organization Journal,**13*(2), Article 100095.32015785

[CR60] Doko, J., Tchabi, F. L. Y. A., Kenali, H. I., Tossa Dognon, D. A., Alia, M. H. B., Zanou, R. A., Yedomonhan, H., Akoègninou, A., & Tossou, G. M. (2023). *Contenu pollinique de l’atmosphère de la ville de Cotonou (Bénin): **C**as des plantes ornementales *[Pollen content of the atmosphere in the city of Cotonou (Benin): Case of ornamental plants]. *Revue Française D’allergologie,**63*(7), Article 103732. 10.1016/j.reval.2023.103732

[CR61] Durham, O. C. (1946). The volumetric incidence of atmospheric allergens. *Journal of Allergy,**17*, 79–86. 21019999 10.1016/0021-8707(46)90025-1

[CR62] Dwarakanath, D., Milic, A., Beggs, P. J., Wraith, D., & Davies, J. M. (2024). A global survey addressing sustainability of pollen monitoring. *World Allergy Organization Journal,**17*(12), Article 100997. 10.1016/j.waojou.2024.100997 39634514 10.1016/j.waojou.2024.100997 PMC11612360

[CR63] ELHassani, L., Boullayali, A., Janati, A., Achmakh, L., & Bouziane, H. (2022). Aerobiological study of airborne pollen in Tétouan (NW of Morocco): Diversity, intensity and calendar. *Aerobiologia,**38*(4), 483–499.

[CR64] Emmerson, K. M., Smith, E., Ebert, E., Milic, A., Vicendese, D., Lampugnani, E. R., Erbas, B., Medek, D. E., Huete, A., Beggs, P., Katelaris, C. H., Haberle, S. G., Newbigin, E., & Davies, J. M. (2022). Evaluation of the performance of short-term curated daily airborne grass pollen forecasts in diverse biogeographical regions during the AusPollen Partnership project 2016–2020. *Atmospheric Science,**15*, Article 100183.

[CR65] Essien, B. C., Adamu, M., Tsoho, S. B., & Ibrahim, G. A. (2024). Aeropalynomorphs, environmental and public health assessment of Mai Mustapha Aliyu International College of Health Science and Technology, Biu, Borno State, Nigeria. *International Journal of Innovative Healthcare Research*, 12(2), 59–74. ISSN: 2354–2950.

[CR66] Esterhuizen, N., Berman, D. M., Neumann, F. H., Ajikah, L., Quick, L. J., Hilmer, E., Van Aardt, A., John, J., Garland, R., Hill, T., Finch, J., Hoek, W., Bamford, M., Seedat, R. Y., Manjra, A. I., & Peter, J. (2023). The South African pollen monitoring network: Insights from 2 years of national aerospora sampling (2019–2021). *Clinical and Translational Allergy,**13*(11), Article e12304. 10.1002/clt2.12304 38006379 10.1002/clt2.12304 PMC10620116

[CR186] Ezike, D. N., Nnamani, C. V., Ogundipe, O. T., & Adekanmbi, O. H. (2016). Airborne pollen and fungal spores in Garki, Abuja (north-Central Nigeria). *Aerobiologia, 32*(4), 697–707. 10.1007/s10453-016-9443-5 PMC5106507 27890967

[CR67] Ezikanyi, D. N., Sakwari, G., & Nnamani, C. V. (2018). Aeroallergens in North-Central Nigeria. *Allergologia Et Immunopathologia,**46*(6), 599–606. 30055844 10.1016/j.aller.2018.03.008

[CR69] Farooq, Q., Oteros, J., & Galán, C. (2025). Advancing in the pollen frontier: A comprehensive evaluation and meta-analysis of automatic pollen monitoring systems. *Aerobiologia,**41*, 527–546. 10.1007/s10453-025-09865-x

[CR70] Filali Ben Sidel, F., Bouziane, H., Trigo, M. M., El Haskouri, F., Bardei, F., Redouane, A., Kadiri, M., Riadi, H., & Kazzaz, M. (2015). Airborne fungal spores of Alternaria, meteorological parameters and predicting variables. *International Journal of Biometeorology*; 59(3):339–46. https://doi/org/10.1007/s00484-014-0845-1 10.1007/s00484-014-0845-1 24844880

[CR71] Filali Ben Sidel, F., Bouziane, H., Trigo, MdelM., El Haskouri, F., & Kadiri, M. (2017). Fungal spores of *Cladosporium* in the air of Tetouan: Meteorological parameters and forecast models. *International Journal of Environmental Sciences & Natural Resources, 3*(2), Article 555609. 10.19080/IJESNR.2017.03.555609

[CR72] Fredoux, A., & Maley, J. (1996). Le contenu pollinique de l’atmosphère dans les forêts du sud Cameroun près de Yaoundé: résultats préliminaires. In *Symposium ‘‘Dynamique along terme des ecosystemes forestiers intertropicaux’’, Paris, 20* (pp. 139–148).

[CR73] Garba, S. A., Hassan, A. M., & Abubakar, M. (2022). Aeromycological studies in tropical West Africa: Seasonal dynamics and health implications. *Environmental Monitoring and Assessment,**194*, 734.36068442

[CR74] George, M. E., Gaitor, T. T., Cluck, D. B., Henao-Martínez, A. F., Sells, N. R., & Chastain, D. B. (2025). The impact of climate change on the epidemiology of fungal infections: implications for diagnosis, treatment, and public health strategies. *Therapeutic Advances in Infectious Disease,**12*, 1–18. 10.1177/20499361251313841 10.1177/20499361251313841 PMC11815821 39944519

[CR75] Gharbi, D., Berman, D., Neumann, F. H., Hill, T., Sidla, S., Cillers, S. S., Staats, J., Esterhuizen, N., Ajikah, L., Moseri, M. E., Quick, L. J., Hilmer, E., Van Aardt, A., John, J., Garland, R., Finch, J., Hoek, W., Bamford, M., Seedat, R. Y., … Peter, J. (2024). Ambrosia (ragweed) pollen — A growing aeroallergen of concern in South Africa. *World Allergy Organization Journal,**17*(12), Article 101011. 10.1016/j.waojou.2024.101011 39698164 10.1016/j.waojou.2024.101011 PMC11652763

[CR76] Gharbi, D., Neumann, F. H., Cilliers, S., Esterhuizen, N., Ajikah, L., Quick, L. J., Hilmer, E., Van Aardt, A., John, J., Garland, R., Hill, T., Finch, J., Hoek, W., Bamford, M., Seedat, R. Y., Manjra, A. I., & Peter, J. (2023a). Allergenic tree pollen in Johannesburg and Cape Town as a public health risk: Towards a sustainable implementation framework for South African cities. *Discover Sustainability,**4*(1), 32. 10.1007/s43621-023-00151-9

[CR79] Gharbi, D., Vanker, A., Garland, R. M., & Peter, J. (2023b). The intersection between air quality aerobiology and asthma in South Africa-could green spaces help? *Current Allergy & Clinical Immunology,**36*(3), 2–6.

[CR78] Gharbi, D., Neumann, F. H., Staats, J., McDonald, M., Linde, J., Mmatladi, T., Podile, K., Piketh, S., Burger, R., Garland, R. M., Bester, P., Lebre, P. H., & Ricci, C. (2025a). Prevalence of aeroallergen sensitization in a polluted and industrialized area: A pilot study in South Africa’s Vaal Triangle. *Environmental Monitoring and Assessment,**197*(1), 287. 10.1007/s10661-025-13718-y 39945937 10.1007/s10661-025-13718-y PMC11825541

[CR77] Gharbi, D., Neumann, F. H., Podile, K., McDonald, M., Linde, J., Frampton, M., Liebenberg, J. L., Cilliers, S., Mmatladi, T., Nkosi, P., Paledi, K., Piketh, S., Staats, J., Burger, R. P., Havenga, H., Garland, R. M., Bester, P., Lebre, P. H., & Ricci, C. (2025b). Exposure to outdoor aerospora and associated respiratory health risks among adults in Potchefstroom, North-West province. *South Africa. Frontiers in Allergy,**6*, 1568669. 10.3389/falgy.2025.1568669 40302853 10.3389/falgy.2025.1568669 PMC12037600

[CR80] Gharnaout, M. (2007). Corrélation entre comptes polliniques et symptomatologie respiratoire allergique au niveau de la ville d’ALGER. [PhD Thesis, Alger]. https://www.ccdz.cerist.dz/admin/notice.php?id=00000000000000214881000000

[CR81] Gharnaout, M., Bencharif, N., Abdelaziz, R., & Douagui, H. (2009). Calendrier pollinique de la ville d’Alger. *Revue des Maladies Respiratoires*, *1495*(10001), 5‑154.

[CR82] Grewling, Ł, Ribeiro, H., Antunes, C., Apangu, G. P., Çelenk, S., Costa, A., Eguiluz-Gracia, I., Galveias, A., Gonzalez Roldan, N., Lika, M., Magyar, D., Martinez-Bracero, M., Ørby, P., O’Connor, D., Penha, A. M., Pereira, S., Pérez-Badia, R., Rodinkova, V., Xhetani, M., … Skjøth, C. A. (2023). Outdoor airborne allergens: Characterization, behavior and monitoring in Europe. *Science of the Total Environment,**905*, Article 167042. 37709071 10.1016/j.scitotenv.2023.167042

[CR83] Grobler, C. H., Bredenkamp, G. J., & Brown, L. R. (2006). Primary grassland communities of urban open spaces in Gauteng, South Africa. *South African Journal of Botany,**72*, 367–377.

[CR84] Hadj Hamda, S., Ben Dhiab, A., Msallem, M., & Larbi, A. (2019). Comparative study of airborne pollen from the northern to the southern of Tunisia. *Asian Journal of Biology,**8*(3), 1–8. 10.9734/ajob/2019/v8i330063

[CR86] Haskouri, F. E., Bouziane, H., Del Mar Trigo, M., Kadiri, M., & Kazzaz, M. (2016). Airborne ascospores in Tetouan (NW Morocco) and meteorological parameters. *Aerobiologia,**32*(4), 669–681. 10.1007/s10453-016-9440-8

[CR192] Hawke, P. R., & Meadows, M. E. (1989). Winter airspora spectra and meteorological conditions in Cape Town, South Africa. *Grana, 28*(3), 187–192.

[CR87] Hayoun, I., El Haskouri, F., & Bouziane, H. (2024). Airborne fungal spores in Tétouan (NW of Morocco), sporal calendar and meteorological parameters. *Revue Française D’allergologie,**64*(6), Article 104146.

[CR88] Hayoun, I., Sidel, F. F. B., Raissouni, I., & Bouziane, H. (2026). *Alternaria* and *Cladosporium* fungal spore concentrations in the atmosphere of Tétouan: Relationships with meteorological parameters and forecasting models. *Aerobiologia,**42*(1), 19. 10.1007/s10453-026-09906-z

[CR89] Humbert, H. (1955). Les territoires phytogéographiques de Madagascar. Colloques internationaux de CNRS LIX : les divisions écologiques du monde. Année biologique : 3ème Sér.31 :329–448

[CR90] Ibigbami, T. & Adeonipekun, A. (2020). Comparative aeropalynology of two communities in Lagos State, southwestern Nigeria. *Notulae Scientia Biologicae,* 12(3), 729–740. 10.15835/nsb12310768

[CR91] Ige, O. E., & Ayeni, O. R. (2019). The study of airborne pollen and spores in Akoko South West environment of Ondo State. *Nigeria. Science Research Annals,**9*(2), 1–9.

[CR92] Ihaddaden, A., Velázquez, E., Rey-Benayas, J.M., & Kadi-Hanifi, H. (2013). Climate and vegetation structure determine plant diversity in Quercus ilex woodlands along an aridity and human-use gradient in Northern Algeria, Flora - Morphology, Distribution, Functional Ecology of Plants, Volume 208, Issue 4, Pages 268–284, IUCN/PACO (2016).

[CR189] Ismail, M. A., Chebon, S. K., & Nakamya, R. (1999). Preliminary surveys of outdoor and indoor aeromycobiota in Uganda. *Mycopathologia, 148*, 41–51. 10.1023/a:1007195708478 11086484

[CR93] Jäckle, H., Rudner, M., & Deil, U. (2013). Density, Spatial Pattern and Relief Features of Sacred Sites in Northern Morocco. *Landscape Online*, 32. 10.3097/LO.20133**2**

[CR94] Janati, A., Bouziane, H., Trigo, M. M., Kadiri, M., & Kazzaz, M. (2017). Poaceae pollen in the atmosphere of Tétouan (NW Morocco): Effect of meteorological parameters and forecast of daily pollen concentration. *Aerobiologia,**33*(4), 517–528.

[CR95] Jury, M. R. (2016). Summer climate of Madagascar and monsoon pulsing of its vortex. *Meteorology and Atmospheric Physics,**128*(1), 117–129. 10.1007/s00703-015-0401-5

[CR96] Kalisa, E., Archer, S., Nagato, E., Bizuru, E., Lee, K., Tang, N., Pointing, S., Hayakawa, K., & Lacap-Bugler, D. (2019). Chemical and biological components of urban aerosols in Africa: Current status and knowledge gaps. *International Journal of Environmental Research and Public Health,**16*(6), 941. 10.3390/ijerph16060941 30875989 10.3390/ijerph16060941 PMC6466367

[CR97] Kalisa, W., Igbawua, T., Henchiri, M., Ali, S., Zhang, S., Bai, Y., & Zhang, J. (2019). Assessment of climate impact on vegetation dynamics over East Africa from 1982 to 2015. *Scientific Reports.,**9*, 16865. 10.1038/s41598-019-53150-0 31727960 10.1038/s41598-019-53150-0 PMC6856068

[CR98] Kemabonta, K. A., Adeonipekun, P. A., Adebayo, M. B., Anumudu, P., & Adeniyi, T. A. (2018). Aerobiology of insect parts in Ayetoro, Ota, Southwest, Nigeria. *Annals of West University of Timişoara, Series of Biology,**21*(1), 57–66.

[CR99] Kiared, G., Bessedik, M., & Riding, J. B. (2016). The aeropalynology of Es-Senia airport, Oran, northwest Algeria. *Palynology,**41*(1), 121–131.

[CR188] Kiprop, V., Nyamache, A. K., Njerwana, S., & Bii, C. C. (2024). Fungal spore air pollution in selected environments in Nairobi, Kenya. *Environmental Pollutants and Bioavailability, 36*(1), 2386168. 10.1080/26395940.2024.2386168

[CR100] Klopper, R. R., Crouch, N. R., Smith, G. F., & van Wyk, A. E. (2020). A synoptic review of the aloes (Asphodelaceae, Alooideae) of KwaZulu-Natal, an ecologically diverse province in eastern South Africa. *PhytoKeys,**142*, 1–88. 32210671 10.3897/phytokeys.142.48365 PMC7082394

[CR101] Korteby-Becila, H. (1987). Contribution à l'étude du composant pollinique de l’air d’Alger. Thèse Doctorale en Sciences Médicales, Institut National d’Enseignement supérieur en Sciences Médicales, Algiers, 274 p.

[CR102] Krzywinski, K. (1977). The Tauber pollen trap: A discussion of its usefulness in pollen deposition studies. *Grana,**16*(3), 147–148.

[CR103] Larrey, E. K., Laryea, J. N. A., Kpordze, S. W., & Saba, C. K. S. (2020). Microbial load of indoor airborne bacteria and fungi in a teaching hospital in Ghana. *African Journal of Microbiology Research,**14*(3), 100–105. 10.5897/AJMR2020.9297

[CR104] Lézine, A.-M., Ivory, S. J., & Braconnot, P. (2019). Vegetation dynamics and pollen diversity in Central African tropical forests. *Quaternary Science Reviews,**211*, 1–16.

[CR106] Lucas, R. W., & Bunderson, L. (2024). A review of pollen counting networks: From the nineteenth century into the twenty-first century. *Current Allergy and Asthma Reports,**24*(1), 1–9. 38153610 10.1007/s11882-023-01119-5

[CR107] Matuvhunye, T., Berman, D. M., Esterhuizen, N., Razafimanantsoa, A. H. I., Neumann, F. H., Gharbi, D., Podile, K., Mmatladi, T., Langa, B., Moseri, M. E., Ajikah, L., Effiom, A., Ndlovu, N., Quick, L. J., Hilmer, E., Guscott, M., Davids, S., Van Aardt, A. C., Linde de Jager, J. C., et al. (2026). Five years of national airborne pollen monitoring in South Africa: Biome-specific calendars to inform allergy diagnosis and prevention. *Aerobiologia,**42*, 23. 10.1007/s10453-026-09909-w 42004049 10.1007/s10453-026-09909-w PMC13083431

[CR108] Mbatchou Ngahane, B.H., Noah, D., Nganda Motto, M. M., Mapoure Njankouo, Y., & Njock, L. R. (2016) Sensitization to common aeroallergens in a population of young adults in a sub-Saharan Africa setting: a cross-sectional study. *Allergy Asthma Clin Immunol* 12, 1 (2016). 10.1186/s13223-015-0107-8 10.1186/s13223-015-0107-8 PMC4700563 26734065

[CR109] Mbugi, E. V., & Chilongola, J. O. (2010). Allergic disorders in Africa and Africans: Is it primarily a priority? *World Allergy Organization Journal,**3*(5), 175–181. 10.1097/WOX.0b013e3181e1976c 23268429 10.1097/WOX.0b013e3181e1976c PMC3488896

[CR193] Moat, J., & Smith, P. (2007). Atlas of the vegetation of Madagascar. Royal Botanic Gardens, Kew, Richmond, UK.

[CR111] Mohamed, M.F., Refaat, M.M., Melek, N.A., Ahmed, E. A., Noor Aldin, N. M., & Abdel Latif, O. M. (2022). Pollen sensitization among Egyptian patients with respiratory allergic diseases.*The Egyptian Journal of Immunology,**29*(4), 01–11. 36197149

[CR112] Moitra, S., & Kavitha, B. (2024). Aerobiology for Clinicians. In *Textbook of Diagnostic and Therapeutic Procedures in Allergy* (pp. 361–373). CRC Press.

[CR113] Mucina, L, & Rutherford, M.C. (2006). The Vegetation of South Africa, Lesotho and Swaziland; Strelitzia 19. South African Biodiversity Institute, Pretoria. 807pp. ISBN-13: 978–1–919976–21–1.

[CR114] Mucina, L., & Rutherford, M. C. (Eds.). (2024). *Zonal Biomes of Southern Africa* (Biome Ecology, Vol. 2). Springer Nature Switzerland AG.

[CR115] Myers, N., Mittermeier, R. A., Mittermeier, C. G., et al. (2000). Biodiversity hotspots for conservation priorities. *Nature,**403*, 853–858. 10706275 10.1038/35002501

[CR117] Necib, A., & Boughediri, L. (2016). Airborne pollen in the El-Hadjar town (Algeria NE). *Aerobiologia,**32*(2), 277–288.

[CR118] Neumann, F. H., Gharbi, D., Ajikah, L., Scott, L., Cilliers, S., Staats, J., Berman, D., Moseri, M. E., Podile, K., Ndlovu, N., Mmatladi, T., & Peter, J. (2025). Ecological and allergenic significance of atmospheric pollen spectra from a Grassland-Savanna ecotone in North West province. *South Africa. Palynology,**49*(2), 2411234. 10.1080/01916122.2024.2411234

[CR182] Nicholson, S. E. (2013). The West African Sahel: A review of recent studies on the rainfall regime and its interannual variability. *International Scholarly Research Notices, 2013*(1), 453521. 10.1155/2013/453521

[CR119] Njokuocha, R. C. (2006). Airborne pollen grains in Nsukka. *Nigeria. Grana,**45*(1), 73–80. 10.1080/00173130600555797

[CR120] Njokuocha, R. C., Agwu, C. O. C., & Okezie, C. E. A. (2017). Effects of weather conditions on selected airborne fungal spores in the southern part of the state of Enugu. *Nigeria. Grana,**56*(4), 263–272. 10.1080/00173134.2016.1248859

[CR121] Okwong, J. W., Adekanmbi, O. H., & Ajikah, L. B. (2019). Palynological analysis of spider webs from Lagos State, Southwestern Nigeria. *International Journal of Botany Studies,**4*(3), 82–87.

[CR122] Olaniyan, T., Dalvie, M. A., Röösli, M., Naidoo, R. N., Künzli, N., de Hoogh, K., Beram, D., Parker, B., Leaner, J., & Jeebhay, M. F. (2020). Short term seasonal effects of airborne fungal spores on lung function in a panel study of schoolchildren residing in informal settlements of the Western Cape of South Africa. *Environmental Pollution,**260*, Article 114023. 32018199 10.1016/j.envpol.2020.114023

[CR124] Ordman, D. (1945). Cypress pollinosis in South Africa. *South African Medical Journal,**19*, 142–153.

[CR125] Ordman, D. (1963). The air-borne fungi in Johannesburg—a second five-year survey: 1955–1959. *South African Medical Journal,**37*(13), 325–328. 13940281

[CR126] Ordman, D. (1964). The regional aspects of respiratory allergy in South Africa. *South African Medical Journal,**38*, 369–372. 14159756

[CR127] Ordman, D., & Etter, K. G. (1956). The airborne fungi in Johannesburg—a five-year survey as a basis for the study of fungus allergy in South Africa. *South African Medical Journal,**30*(44), 1054–1058. 13380575

[CR128] Orijemie, E. A. (2025). *Prof. Margaret Adebisi Sowunmi (1939–2025).* PALYNOS: Newsletter of the International Federation of Palynological Societies, 48(2).

[CR129] Orlandi, F., Oteros, J., Aguilera, F., Dhiab, A. B., Msallem, M., & Fornaciari, M. (2014). Design of a downscaling method to estimate continuous data from discrete pollen monitoring in Tunisia. *Environmental Science: Processes & Impacts,**16*(7), 1716–1725. 24824947 10.1039/c4em00153b

[CR130] Oteros, J., Orlandi, F., García-Mozo, H., Aguilera, F., Ben Dhiab, A., Bonofiglio, T., Abichou, M., Ruiz-Valenzuela, L., Del Trigo, M. M., Díaz de la Guardia, C., Domínguez-Vilches, E., Msallem, M., Fornaciari, M., & Galán, C. (2014). Better prediction of Mediterranean olive production using pollen-based models. *Agronomy for Sustainable Development,**34*, Article 685694.

[CR131] Oteros, J., Sofiev, M., Smith, M., Clot, B., Damialis, A., Prank, M., Werchan, M., Wachter, R., Weber, A., Kutzora, S., Heinze, S., Herr, C. E. W., Menzel, A., Bergmann, K. C., Traidl-Hoffmann, C., Schmidt-Weber, C. B., & Buters, J. T. M. (2019). Building an automatic pollen monitoring network (ePIN): Selection of optimal sites by clustering pollen stations. *Science of the Total Environment,**688*, 1263–1274. 10.1016/j.scitotenv.2019.06.131 31726556 10.1016/j.scitotenv.2019.06.131

[CR132] Patra, H. R., & Muchie, M. (2021). Scientific and technical productivity of African countries: What Scopus and WIPO Patentscope data tell us. *Journal of Scientometric Research,**10*(3), 355–365.

[CR133] Peck, R. M. (1972). Efficiency test on the Tauber trap used as a pollen sampler in turbulent water flow. *New Phytologist,**71*, 187–198. 10.1111/j.1469-8137.1972.tb04827.x

[CR135] Potter, P. C., Berman, D. M., Toerien, M. A., & Weinberg, E. G. (1991). Clinical significance of aero-allergen identification in the Western Cape. *South African Medical Journal,**79*, 80–84. 1989093

[CR136] Rabarisoa, N. (2007). *Emissions polliniques, phénologie de la floraison, facteurs climatiques et épidémiologie des allergies à Antsirabe* (p. 94p). D.E.A. Palynologie Appliquée, Univ. Antananarivo.

[CR137] Raissouni, I., Achmakh, L., Boullayali, A., El Gharbi, M., & Kazzaz, A. (2024a). Forecast models for start and peak dates of Poaceae pollen season in Tétouan (NW Morocco) using multiple regression analysis. *International Journal of Biometeorology,**68*, 2215–2225. 39060702 10.1007/s00484-024-02739-w

[CR138] Raissouni, I., Achmakh, L., Boullayali, A., El-Khattabi, J., Hassoun, M., & Bouziane, H. (2025). Thermal requirements and forecasting the start of the plane tree pollen season in Tétouan (NW Morocco). *International Journal of Biometeorology*. 10.1007/s00484-025-02939-y 10.1007/s00484-025-02939-y 40327071

[CR139] Raissouni, I., Boullayali, A., Recio, M., & Bouziane, H. (2024b). Variations, trends and forecast models for the airborne *Olea europaea* pollen season in Tétouan (NW Morocco). *International Journal of Biometeorology,**68*(12), 2613–2625. 39235597 10.1007/s00484-024-02772-9

[CR140] Rajeriarison, C. (1984). *Influences des formations végétales Malgaches et des principaux facteurs climatiques dans la composition des flux polliniques atmosphériques de la région de Tananarive (MADAGASCAR) au cours de 3 cycles annuels (1979 1980 et 1981)* (p. 150p). Thèse de Doctorat d’état. Univ.

[CR141] Rakotomamonjy, R. (2016). Étude des espèces végétales allergisantes à Mahajanga et analyse de leurs impacts sur la santé publique. Université de Mahajanga. 65p.

[CR142] Rakotonirina, S. (2008). *Composition pollinique de l’atmosphère et calendrier pollinique d’Antananarivo durant le cycle annuel 2005–2006* (p. 94p). D.E.A. Palynologie Appliquée, Univ. Antananarivo.

[CR143] Rakotoson, H. (2007). *Émissions polliniques, phénologie de la floraison, facteurs climatiques et épidémiologie des allergies à Ambatondrazaka* (p. 117). D.E.A. Palynologie Appliquée, Faculté des Sciences, Université d’Antananarivo.

[CR144] Ramavovololona, P. (1986). Recherche sur les émissions polliniques atmosphériques des formations végétales de la région de Majunga. Morphologie des principaux types polliniques. Mise en évidence des caractéristiques régionales, des spectres polliniques de Majunga. Thèse de Doctorat de 3e cycle de Sciences Biologiques Appliquées, option Écologie Végétale. Univ. Antananarivo, Fac.des Sciences, 171 p.

[CR194] Ramavovololona, P. (1998). Étude palynologique et immunoallergique de 8 espèces de graminées et d’une espèce d’Asteraceae commune de Madagascar. Doctoral thesis (Doctorat d’État), Université d’Antananarivo, Madagascar. 171 pp.

[CR145] Ramavovololona, S. H., Ramamonjisoa, Z. R., Andrianarisoa, A. C. F., Rakotoarimanana, V. M., Sutra, J. P., & Peltre, G. (2013). Allergy to Pollen of Six Common Grasses in the Highlands of Madagascar. *Current Allergy & Clinical Immunology,**26*(4), 184–192. 10.10520/EJC148493

[CR146] Randrianandraina, P. M., Solo C. E., Andriambelo R. H., Razafimahatratra M.J.J., Ramilison H. E., Mamiharilala M. C., & Rakotoarisoa, R. H. N. (2022). “Implication du pollen de canne à sucre dans les manifestations de la rhinite allergique: étude cas-témoins.” *Pan African Medical Journal* 41. 10.11604/pamj.2022.41.133.27897. 10.11604/pamj.2022.41.133.27897 PMC9034559 35519171

[CR147] Randrianjafy, I. (2018). Pollen des plantes à fleurs et/ou mellifères de la ville de Mahajanga. 63p.

[CR148] Rasolofonirina, F. V. (2025). *Calendrier pollinique suivant la période de floraison des plantes allergisantes dans l’atmosphère de la ville de Mahajanga* (p. 66). Université de Mahajanga.

[CR149] Razafimanantsoa, A. H. I., Bond, W. J., & Gillson, L. (2025). Vegetation Dynamics and Drivers of Change in the Central Highlands of Madagascar during the Last 6300 Years: Pre- and Post-Human Settlement. *The Holocene,**35*(4), 460–470. 10.1177/09596836241307295

[CR150] Razafimanantsoa, A. H. I., & Razanatsoa, E. (2024). Modern Pollen Rain Reveals Differences across Forests, Open and Mosaic Landscapes in Madagascar. *Plants, People, Planet,**6*(3), 729–742. 10.1002/ppp3.10487

[CR151] Razafimelison, H. F. (2008). *Le contenu pollinique de l’atmosphère d’Antananarivo : Relation avec les facteurs climatiques et les données épidémiologiques* (p. 96p). DEA Palynologie Appliquée Végétale, Univ. Antananarivo, Fac. Sciences.

[CR152] Razanatsoa, E., Gillson, L., Virah-Sawmy, M., & Woodborne, S. (2022). Synergy between Climate and Human Land-Use Maintained Open Vegetation in Southwest Madagascar over the Last Millennium. *The Holocene,**32*(1–2), 57–69. 10.1177/09596836211041731

[CR153] Refaat, M. M., Melek, N. A. E., Ahmed, E. E., Abdellatif, O. M., Mohamed, M. F., & Noor Al-Din, N. M. (2021). The most prevalent allergenic airborne pollens among Egyptian patients with respiratory allergy. *QJM: An International Journal of Medicine, 114* (Supplement_1), hcab100.143. 10.1093/qjmed/hcab100.143

[CR154] Regional Assessment on Ecosystem-based Disaster Risk Reduction and Biodiversity in West and Central Africa. A report for the Resilience through Investing in Ecosystems – knowledge, innovation and transformation of risk management (RELIEF Kit) project. Ouagadougou, Burkina Faso: IUCN. 58pp.

[CR155] Roffe, S., Ajikah, L., John, J., Garland, R. M., Lehtipalo, K., & Bamford, M. K. (2025). High aerospora levels and associated atmospheric circulation patterns: Pretoria, South Africa. *International Journal of Biometeorology,**69*, 2441–2456. 10.1007/s00484-024-02781-8 39333404 10.1007/s00484-024-02781-8 PMC12540555

[CR156] Rutherford, M. C., & Westfall, R. H. (1986). The biomes of Southern Africa — an objective categorization. *Memoirs of the Botanical Survey of South Africa,**51*, 1–98.

[CR190] Savina, K. A., Kuzmicheva, E. A., Severova, E. E., Khasanov, B. F., Girmay, W. W., Nemomissa, S., Krylovich, O. A., & Savinetsky, A. B. (2025). Modern pollen rain in the Bale Mountains (Ethiopia) along an elevational gradient. *Biology Bulletin, 52*(5), 542–556. 10.31857/S1026347025050052

[CR157] Sayah, W., Guermache, I., Berkane, I., Boudiaf, N., & Ait-Khaled, N. (2021). Profil clinique et allergologique des pollinoses dans la région d’Alger. *Revue Algérienne d’Allergologie*, 6(1), 17–22.

[CR158] Sofiev, M., Buters, J., Tummon, F., et al. (2023). Designing an automatic pollen monitoring network for direct usage of observations to reconstruct the concentration fields. *Science of the Total Environment,**900*, Article 165800. 10.1016/j.scitotenv.2023.165800 37595925 10.1016/j.scitotenv.2023.165800

[CR160] Straka, H. (1967). Palynologia Madagassica et Mascarenica. Fam. 50 et 59 bis. *Pollen Et Spores,**8*, 241–264.

[CR161] Straka, H., & Friedrich, B. (1982). Palynologia Madagassica et Mascarenica. Fam. 147 à 154. *Pollen Et Spores,**24*, 54–71.

[CR162] Straka, H., & Friedrich, B. (1983). Palynologia Madagassica et Mascarenica. Fam. 128 à 146. *Pollen Et Spores,**44*, 8–93.

[CR163] Straka, H., & Friedrich, B. (1984). Palynologia Madagassica et Mascarenica. Fam. 17 à 49. *Pollen Et Spores,**49*, 6–89.

[CR164] Straka, H., & Friedrich, B. (1988). Palynologia Madagassica et Mascarenica. Fam. 65 à 97. *Pollen Et Spores,**61*, 6–117.

[CR165] Straka, H., & Simon, A. (1967). Palynologia Madagassica et Mascarenica. Fam. 122 à 166. *Pollen Et Spores,**9*, 428–466.

[CR166] Tadross, M., Randriamarolaza, L., Rabefitia, Z., Ki, & Yip Z. (2008). Climate change in Madagascar: recent past and future. Washington, DC: World Bank. Available from. http://www.mediagrapher.org/gripweb/sites/default/files/disaster_risk_profiles/Madagascar%20Climate%20Report.pdf

[CR167] Taia, W. K., Ibrahim, M. I., & Bassiouni, E. M. (2019). Study of the airborne pollen grains in Rosetta. *Egypt. International Journal of Advanced Research and Publications,**3*(3), 122–129.

[CR168] Tchabi, F. L., Tossou, G. Monique, Zanou, R. Adéline, Akoègninou, Akpovi, & Akpagana, Koffi. (2017b)*.* Caractérisation Du Contenu Pollinique De L’atmosphère De La Commune d’Abomey-Calavi. De 2015 À 2017. *European Scientific Journal* 13 (30), 417–440

[CR169] Tchabi, F. L., Tossou, G. M., Akoegninou, A., & Trigo, M. M. (2017a). Étude aéropalynologique de la commune d’Abomey-Calavi (Bénin) au cours de la grande saison des pluies. *Revue Française D’allergologie,**57*(4), 308–316.

[CR191] Tesfamichael, S. G., Shiferaw, Y. A., & Phiri, M. (2022). Monthly geographically weighted regression between climate and vegetation in the Eastern Cape Province of South Africa: Clustering pattern shifts and biome-dependent accuracies. *Scientific African, 18*, e01423.

[CR170] Tossou, G. M., Chabi, L. F., Akoègninou, A., Ballouche, A., & Akpagana, K. (2016). Analyse pollinique de l’atmosphère du campus d’Abomey-Calavi (Bénin). *Revue Française D’allergologie,**56*(2), 65–75.

[CR171] Tummon, F., Adams-Groom, B., Antunes, C. M., Bruffaerts, N., Buters, J., Cariñanos, P., Celenk, S., Choël, M., Clot, B., Cristofori, A., Crouzy, B., Damialis, A., Rodríguez Fernández, A., Fernández González, D., Galán, C., Gedda, B., Gehrig, R., Gonzalez-Alonso, M., Gottardini, E., de Weger, L. A., et al. (2024). The role of automatic pollen and fungal spore monitoring across major end-user domains. *Aerobiologia,**40*, 57–75. 10.1007/s10453-024-09820-2

[CR172] Van Leuken, J. P. G., Swart, A. N., Droogers, P., van Pul, A., Heederik, D., & Havelaar, A. H. (2016). Climate change effects on airborne pathogenic bioaerosol concentrations: A scenario analysis. *Aerobiologia,**32*, 607–617. 27890966 10.1007/s10453-016-9435-5 PMC5106502

[CR176] Van Wyk, AE. (1996). Biodiversity of the Maputaland Centre. In: Van der Maesen LJG, Van derBurgt XM, Van Medenbach de Rooy JM (Eds) The Biodiversity of African Plants, Kluwer Academic Publishers, Dordrecht, 198–207. 10.1007/978-94-009-0285-5-26

[CR173] Vololona, J., Ramamonjisoa, R. Z., Rasoamanana, E. N., & Ramavovololona, P. (2019). Morphologie pollinique de la flore de la Réserve Spéciale d’Ankarana, Madagascar. *Malagasy Nature,**13*, 1–51.

[CR174] Vorontsova, M. S., Besnard, G., Forest, F., Malakasi, P., Moat, J., Clayton, W. D., Ficinski, P., Savva, G. M., Nanjarisoa, O. P., Razanatsoa, J., Randriatsara, F. O., Kimeu, J. M., Luke, W. R. Q., Kayombo, C., & Linder, H. P. (2016). Madagascar’s grasses and grasslands: Anthropogenic or natural? *Proceedings of the Royal Society B,**283*, 20152262. 10.1098/rspb.2015.2262 26791612 10.1098/rspb.2015.2262 PMC4795014

[CR177] Yafetto, L., & Adator, E. H. (2018). Fungal contaminations of indoor and outdoor air of buildings of the University of Cape Coast. *Ghana. Studies in Fungi,**3*(1), 333–342. 10.5943/sif/3/1/33

[CR179] Yangui, F., Charfi, M. R., Khouani, H., Triki, M., & Abouda, M. (2018). Profils cliniques et allergéniques des pollinoses en Tunisie. *Revue Française d'Allergologie*, *58*(8), 549–555. 10.1016/j.reval.2018.01.008

[CR178] Yangui, F., Khouani, H., Abouda, M., Boussetta, K., Ben Salem, A., & Rouatbi, N. (2015). Changes in pollen sensitization over last twenty years in Tunisia. *European Respiratory Journal,* 46, 59: OA4469. 10.1183/13993003.congress-2015.OA4469

[CR180] Yazidi, A., Nejjari, C., & Bartal, M. (2001). La sensibilisation cutanée aux pollens au Maroc. *Revue des Maladies Respiratoires,**18*, 523–529. 11887770

[CR181] Ybert, J. P. (1980). Le contenu pollinique de l’atmosphère en Côte d’Ivoire et au Tchad. *Grana,**19*(1), 31–46. 10.1080/00173138009424985

